# USP15 mutation associated with autism spectrum disorder alters progenitor fate and neuronal maturation in human brain organoids

**DOI:** 10.1016/j.mocell.2026.100387

**Published:** 2026-07-29

**Authors:** Tae-Hwan Park, In Gyeong Koh, Seoyoung Sung, Hayoon Park, Jieun Kim, Sebin Lee, Yun Jin Lee, Hyunsoo Ko, Jae Hyun Han, Guiyoung Bong, Hee Jeong Yoo, Jaesang Kim, Joon-Yong An, Ji Yeoun Lee

**Affiliations:** 1 Department of Anatomy and Cell Biology, Seoul National University College of Medicine, Seoul, Republic of Korea; 2 Department of Translational Medicine, Seoul National University College of Medicine, Seoul, Republic of Korea; 3 L-HOPE Program for Community-Based Total Learning Health Systems, Korea University, Seoul, Republic of Korea; 4 Department of Integrated Biomedical and Life Science, Korea University, Seoul, Republic of Korea; 5 National Research Laboratory for Convergence Degradation Biology, Korea University, Seoul, Republic of Korea; 6 Department of Life Science, Ewha Womans University, Seoul, Republic of Korea; 7 School of Biosystem and Biomedical Science, College of Health Science, Korea University, Seoul, Republic of Korea; 8 Department of Biochemistry & Molecular Biology, Faculty of Medicine, University of British Columbia, Vancouver, British Columbia V6T 1Z4, Canada; 9 Terry Fox Laboratory, British Columbia Cancer Research Institute, Vancouver, British Columbia V5Z 1L3, Canada; 10 Department of Psychiatry, Seoul National University Bundang Hospital, Seongnam, Republic of Korea; 11 Department of Psychiatry, Seoul National University College of Medicine, Seoul, Republic of Korea; 12 Division of Pediatric Neurosurgery, Seoul National University Children's Hospital, Seoul, Republic of Korea

**Keywords:** Corticogenesis, Deubiquitinase, Haploinsufficiency, Neurodevelopmental disorders, Transcriptome

## Abstract

De novo variants in the ubiquitin-proteasome pathway are linked to autism spectrum disorder (ASD), yet their functional impact on neurodevelopment remains poorly understood. We investigated *USP15*, a deubiquitinating enzyme with rare damaging variants identified in individuals with ASD, using isogenic human iPSC-derived brain organoids and single-cell transcriptomics. USP15 mutant organoids showed genotype-dependent, progenitor-centered alterations during corticogenesis. Heterozygous organoids modeling haploinsufficiency displayed a shift toward later pseudotime states together with altered maturation and synaptic organization of deep-layer neurons. In contrast, homozygous organoids showed broader phenotypes, including mitotic suppression, aberrant *HOX* gene expression, and stress-response activation. Regulon analysis showed reduced activity of progenitor-associated regulons, including SOX2, NR2F1, and NR2F2, in heterozygous organoids, whereas homozygous organoids exhibited broader changes in transcriptional regulatory networks. Furthermore, *USP15* mutant-associated gene expression patterns were significantly enriched for established ASD risk genes. Comparison with the mouse brain perturbation atlas showed that the transcriptional signature of the *USP15* mutant showed notable overlap with those of *Fezf2* and *Foxp1* mutants, key regulators of deep-layer projection neuron identity. These findings characterize genotype-dependent neurodevelopmental phenotypes associated with reduced USP15 dosage and provide a human neural framework for investigating ASD-relevant developmental mechanisms in the context of a rare ubiquitin-pathway variant.

## Graphical Abstract

**Figure:**
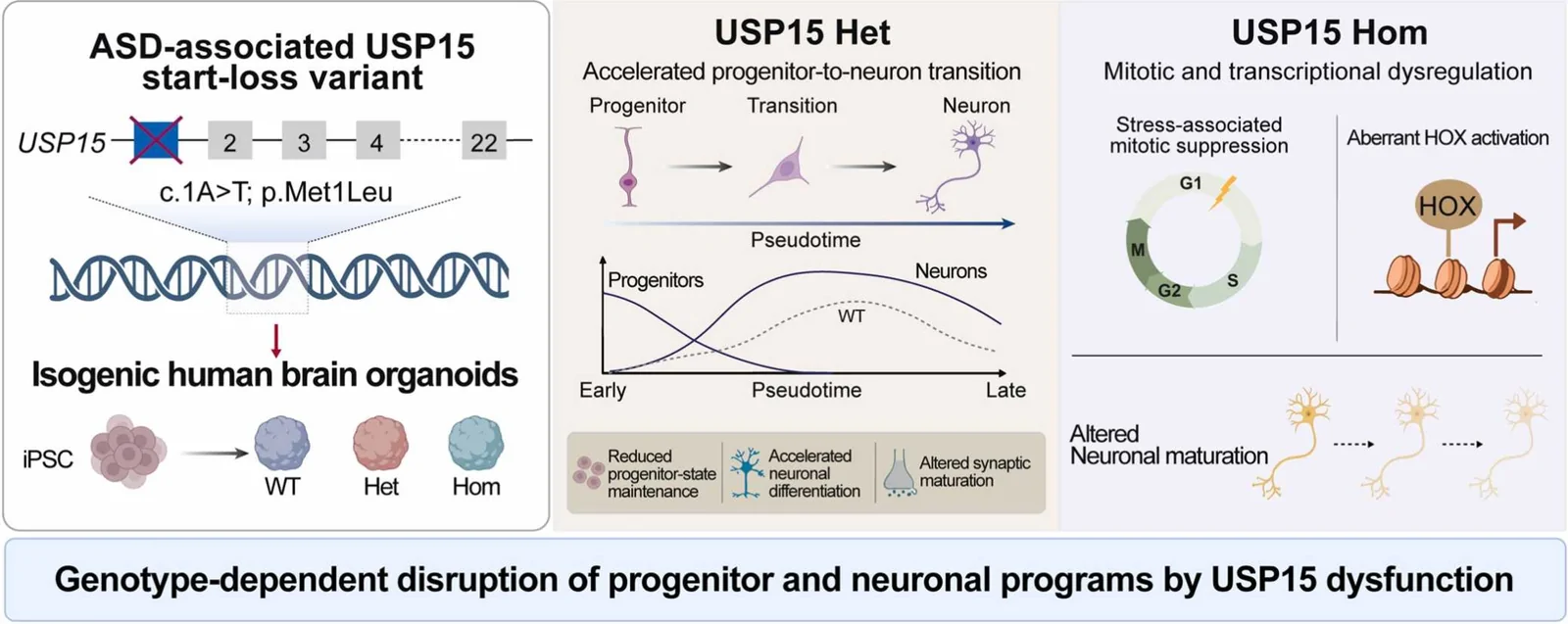

## INTRODUCTION

Autism spectrum disorder (ASD) is a complex neurodevelopmental disorder characterized by genetic heterogeneity (Choi and An, 2021). Over the past decade, large-scale genomic studies have identified hundreds of genes affected by de novo protein-truncating and missense variants (Fu et al., 2022, Satterstrom et al., 2020, Trost et al., 2022), prioritizing high-confidence risk genes replicated across multiple ASD cohorts (Abdi et al., 2023, Al-Mubarak et al., 2017, An et al., 2014, Kim et al., 2024a, Kimura et al., 2022). These risk genes are mainly involved in synaptic function and gene regulation pathways during early corticogenesis (Satterstrom et al., 2020) and have been extensively studied to elucidate their molecular mechanisms (Ben-Shalom et al., 2017, Jung et al., 2018, Kweon et al., 2021). These pathways converge on the mid-fetal cortex, highlighting spatiotemporal specificity in the molecular pathology of ASD (Jeong et al., 2025, Kim and An, 2025, Parikshak et al., 2013, Willsey et al., 2013).

While synaptic and chromatin pathways have been extensively studied, other risk gene-associated pathways remain less characterized in the context of ASD. One such pathway is the ubiquitin-proteasome system, which has been repeatedly implicated in association with ASD. Genes such as *UBE3A* (Yi et al., 2015), *CUL3* (Rapanelli et al., 2021), *USP7* (Fountain et al., 2019), and *RNF8* (Valnegri et al., 2017) encode components of the ubiquitin system and have been involved in ASD. Various de novo damaging variants (protein-truncating or missense with MPC >2) were identified in ubiquitin-pathway genes across large-scale ASD cohorts, totaling 125 variants in ASD cases compared with 28 in unaffected siblings (Fu et al., 2022). Still, the small number of affected individuals per gene limits statistical power to achieve genome-wide significance. For example, our previous study (Kim et al., 2024a) identified damaging variants in *USP15*, a highly loss-of-function intolerant gene (Lek et al., 2016), similar to other ASD genes. However, the small number of identified variants was insufficient to establish gene-level statistical significance, highlighting the need for functional models to investigate the neurodevelopmental consequences associated with individual variants.

Brain organoids provide a powerful platform for examining the functional consequences of damaging variants during early neurodevelopment (Li et al., 2023, Mariani et al., 2015, Paulsen et al., 2022, Sebastian et al., 2023). By recapitulating brain developmental processes, they enable investigation of how early neurogenesis, which is critical for autism neurobiology, is affected by genetic perturbations. Combining brain organoid models with single-cell RNA sequencing enables characterization of cell-type-specific transcriptional differences associated with a defined genetic variant and their distribution along inferred developmental trajectories. This approach is particularly valuable for studying gene haploinsufficiency, the most common genetic state observed in patients with ASD carrying damaging variants. Additionally, the isogenic nature of organoid models allows direct comparison between wild-type and mutant conditions, providing a controlled framework for examining developmental programs associated with specific variants.

In this study, we examined a heterozygous *USP15* start-codon-disrupting variant (c.1A>T; p.Met1Leu) identified in an individual with ASD (Kim et al., 2024a). We used human-induced pluripotent stem cell (hiPSC)-derived cortical organoids, which recapitulate key aspects of early human corticogenesis and neuronal differentiation. We generated isogenic hiPSC lines harboring heterozygous and homozygous *USP15* mutations and profiled brain organoids by single-cell transcriptomics. Our analyses identified genotype-associated differences in progenitor-state maintenance, neuronal maturation, and transcriptional regulatory programs. By placing these cellular and transcriptional features within ASD-relevant gene networks, this study provides a human neural model for investigating neurodevelopmental consequences associated with reduced USP15 dosage.

## MATERIALS AND METHODS

### Human iPSC Maintenance

The hiPSC line #5-1 was derived from epidermal fibroblasts (Lee et al., 2022) and maintained under feeder-free conditions in mTeSR Plus (StemCell Technologies) on Matrigel or vitronectin-coated tissue culture plates. Cells were cultured at 37°C, 5% CO_2_, with daily medium changes. They were passaged every 5 to 7 days using ReLeSR (StemCell Technologies), and experiments were performed with passages ≤30. At banking and prior to differentiation, the line was evaluated for attachment and morphology, with no abnormalities noted when compared across routine passages.

### sgRNA Design and Donor Templates

The single-guide RNA (sgRNA, 5’-ccgcuaguggaagaagaugg-3’) targeting the initial codon of *USP15* was designed using Cas-Designer (http://www.rgenome.net/cas-designer/) and DeepSpCas9 (https://deepcrispr.info/DeepSpCas9/). The protospacer adjacent motif of the sgRNA located proximally was selected for the introduction of NM_001252078.2: p.Met1Leu mutation into the *USP15* gene. Phosphorylated sgRNA oligonucleotides were annealed and cloned into pSpCas9(BB)-2A-GFP (PX458) (Addgene, plasmid #48138) as previously described (Ran et al., 2013). To induce the specific mutation, single-stranded oligo-deoxynucleotide (ssODN) templates were designed. The sequences of the ssODNs are as follows:

ssODN wild type:

5’-GGCGTGCCGCCGCCGTCGCTGCCACCTCCCCTACCGCTAGTGGAAGAAGATGGCGGA

AGGCGGAGCGGCGGATCTGGACACCCAGCGGTCTGACATCGCG-3’,

ssODN mutant:

5’-GGCGTGCCGCCGCCGTCGCTGCCACCTCCCCTACCGCTAGTGGAAGAAGTTGGCGGA

AGGCGGAGCGGCGGATCTGGACACCCAGCGGTCTGACATCGCG-3’.

### Electroporation and Clone Isolation

A total of 10 µg of the PX458-*USP15* plasmid was co-electroporated with 5 µg of each ssODN into 1,000,000 iPS cells (line #5-1) using the Neon Transfection System (Invitrogen, USA). Following transfection, the cells were plated onto Matrigel-coated 60-mm dishes in mTeSR Plus medium supplemented with 10 μM Y-27632. After 72 hours, GFP-positive cells were sorted using a BD FACSAria Fusion (Becton, Dickinson and Company, USA) at the Ewha Fluorescence Core Imaging Center. The sorted cells were then seeded into 100-mm dishes at a clonal density (approximately 1,500 cells per dish) in mTeSR Plus medium supplemented with 10 μM Y-27632. After roughly 7 days of sorting, individual iPSC clones were manually picked and transferred to 48-well plates containing mTeSR Plus medium.

### Genotyping and Off-Target Assessment

Genomic DNA was extracted from the expanded cells for PCR analysis to verify positive clones. PCR analysis and Sanger sequencing were performed using the following primers: forward, 5’-CGAGGAAAGCTAAAACCCGG-3’, reverse, 5’-GCACCTGTCACTAGCGAAAG-3’. To validate the clonality of isolated cells, the PCR products were subcloned using TOPcloner PCR cloning kit (Enzynomics, EZ012S), and at least 20 clones were analyzed by Sanger sequencing.

The web tool Cas-OFFinder (https://www.rgenome.net/cas-offinder/) was used to predict potential off-target sites. Six candidate off-target sites in the genome that had up to 3 base mismatches (Anderson et al., 2015) were amplified and assessed by Sanger sequencing. No sequence alterations were detected at the 6 in silico-predicted off-target loci examined by Sanger sequencing.

### Western Blot

Cells were homogenized in RIPA buffer (50 mM Tris-HCl [pH 8.0], 2 mM EDTA, 150 mM NaCl, 1% NP-40, 0.5% sodium deoxycholate, 2% SDS, and 10 mM sodium fluoride) supplemented with protease and phosphatase inhibitors. Protein concentration was quantified using the Pierce BCA Protein Assay Kit (Thermo Scientific, 23227). Samples were mixed with 4× Laemmli Sample Buffer (GenDEPOT, L1200-001) supplemented with 2-mercaptoethanol, heated at 95°C for 5 minutes, and chilled on ice. A total of 7.5 μg of protein per sample and a Xpert Prestained Protein Marker (GenDEPOT, P8502) were loaded into each lane and separated by 10% SDS-PAGE. After electrophoresis, proteins were transferred onto a 0.45-μm PVDF membrane (Merck Millipore, IPVH00010). The membrane was blocked with 5% skim milk in 1× TBST buffer for 1 hour on an orbital shaker at room temperature and incubated overnight at 4°C with an antibody targeting the carboxy terminus of human USP15 (Cell Signaling, #66310; 1:1,000 dilution in 5% skim milk) with gentle shaking. The membrane was washed 3 times for 10 minutes each in 1× TBST and incubated with an HRP-conjugated anti-rabbit IgG secondary antibody (Cell Signaling, #7074; 1:5,000 dilution in 5% skim milk) for 1 hour at room temperature. After 3 additional 10-minute washes in 1× TBST, protein expression was visualized using SuperSignal West Pico PLUS Chemiluminescent Substrate (Thermo Scientific, 34577) and captured using the ChemiDoc Touch Imaging System (Bio-Rad, USA). For internal control detection, the membrane was rinsed in water and incubated in a mild stripping buffer (15 g of glycine, 1 g of SDS, 10 ml of Tween-20, and deionized water to 1,000 ml; pH 2.2) with agitation for 10 minutes at room temperature. Following stripping, the membrane was reblocked and incubated with a primary antibody against α-tubulin (Sigma-Aldrich, T5168, 1:10,000 dilution in 5% skim milk) and an HRP-conjugated anti-mouse IgG secondary antibody (Cell Signaling, #7076; 1:5,000 dilution in 5% skim milk) as described above.

### RT-qPCR Assay

RT-qPCR assay was performed using RNA extracted from WT, Het, and Hom hiPSC lines with RNeasy Plus Mini Kit (QIAGEN, 74134). About 1μg of total RNA was reverse-transcribed into cDNA using QuantiTect Reverse Transcription Kit QIAGEN, 205311). RT-qPCR assay using SYBR Green qPCR Master Mix (Thermo Scientific, A66732) was performed on the StepOnePlus Real-Time PCR System (Applied Biosystems, USA) following the manufacturer’s protocol. Transcripts were targeted with primers spanning USP15 exons 1 and 2 and normalized to 18S rRNA. The forward primer for USP15 was 5’-GTGGAAGAAGATGGCGGAAG-3’ and the reverse primer was 5’-CGACTAGGTACCAGGTGTCC-3’ the forward primer for 18S rRNA was 5’-CTTAGAGGGACAAGTGGCG-3’ and the reverse primer was 5’-ACGCTGAGCCAGTCAGTGTA-3’. Relative USP15 mRNA levels were calculated from quadruplicate Ct values according to the 2^−ΔΔCt^ method (Livak and Schmittgen, 2001). Statistical analysis was performed to determine significant differences in RNA levels between cell lines using a one-way ANOVA, followed by Holm-Sidak’s post hoc test.

### Cerebral Organoid Generation and Culture

Cerebral organoids were generated from isogenic hiPSC lines (#5-1, *USP15* heterozygous mutant, *USP15* homozygous mutant) using the Cerebral Organoid Kit (StemCell Technologies) according to the manufacturer’s instructions and published protocols (Giandomenico et al., 2021). Briefly, 12,000 dissociated hiPSCs were seeded per well in U-bottom, low-attachment (ultra-low-attachment or equivalent) 96-well plates to form embryoid bodies (EBs). On day 5, EBs were switched to neural induction medium and transferred to low-attachment 24-well plates. On day 7 to 8, EBs exhibiting clear neuroectodermal layers were embedded in Matrigel in expansion medium and moved to low-attachment 6-well plates. On day 14, the Matrigel was removed, and the organoids were maintained in a free-floating culture. From day 10 onward, organoids were maintained in maturation medium on an orbital shaker. Media were refreshed every 2 days, and organoids were maintained to day 107, when samples were harvested for downstream assays. All genotypes were processed in parallel under identical conditions.

### Immunofluorescence Analysis

At predefined time points, cerebral organoids were fixed in 4% paraformaldehyde (PFA) for 3 hours and subjected to sucrose sinking. The organoids were frozen and sectioned for immunofluorescence staining at 20 μm. For immunofluorescence staining, the slides were fixed and permeabilized with a permeabilization solution (554714; BD Cytofix/Cytoperm) for 20 minutes, blocked for 1 hour at room temperature, and then incubated overnight with primary antibodies. Next, the slides were incubated with secondary antibodies labeled with Alexa Fluor 488, 594, or 647 (1:500; Invitrogen) for 2 hours. DAPI (62248; Thermo Fisher) stained cell nuclei. Images were acquired by confocal microscopy (Zeiss LSM 980).

The primary antibodies were as follows: SOX2 (1:250, 3579; Cell Signaling), SOX2 (1:250, AF2018; R&D Systems), DCX (1:250, sc-271,390; Santa Cruz Biotechnology), NeuN (1:100, MAB377; Millipore), MAP2 (1:1000, NB300-213; Novus Biologicals), CTIP2 (1:100, ab28448; Abcam), BRN2 (1:400, sc-393324; Santa Cruz), SYN1 (1:250, S0664; Sigma), and PSD95 (1:250, 51-6900; Invitrogen).

### Image Quantification

For imaging-based quantification, multiple organoids were analyzed per genotype across independent differentiation batches. The number of independent differentiation batches, organoids analyzed per genotype, and ROIs or image fields analyzed per organoid are provided in the corresponding figure legends. Unless otherwise indicated, ROI- or field-level measurements were averaged within each organoid, and the resulting organoid-level mean was used as the unit for statistical analysis.

Organoid cross-sectional areas were measured from bright-field images at days 40, 60, 80, and 100 using ImageJ/Fiji. Statistical comparisons were performed using a linear model including genotype and differentiation batch.

For immunofluorescence-based cell quantification, confocal images were acquired using identical acquisition settings across genotypes within each staining experiment. Marker-positive nuclei or cells were segmented and counted using StarDist (Schmidt et al., 2018), a deep learning-based plug-in in ImageJ/Fiji, with identical segmentation and thresholding parameters applied across genotypes within each staining experiment.

For synaptic puncta analysis, day 100 organoid sections immunostained for MAP2, SYN1, and PSD95 were imaged using a Zeiss LSM 980 confocal microscope with a 63× objective and 3.5× digital zoom. Each image field corresponded to 38.48 × 38.48 μm. Z-stack images were acquired using identical laser and acquisition settings across genotypes. Four or more ROIs were sampled from spatially separated, predefined radial positions when artifact-free MAP2-positive neuronal layer regions were available. ROIs were selected using consistent image-quality criteria, excluding regions with tissue folding, tissue damage, imaging artifacts, or insufficient MAP2-positive neurites. All subsequent dendrite segmentation, puncta detection, colocalization analysis, and normalization procedures were performed using identical parameters across genotypes.

MAP2-positive dendrites were segmented using the SynQuant (Wang et al., 2020) plug-in in ImageJ/Fiji, and MAP2-positive dendrite length was measured from the resulting dendrite mask. SYN1 puncta, PSD95 puncta, and SYN1-PSD95 colocalized puncta were quantified using SynBot (Savage et al., 2024) with identical analysis parameters across all ROIs and genotypes. Puncta densities were calculated as the number of puncta per MAP2-positive dendrite length.

### Preprocessing of Single-Cell RNA-seq Data

Single-cell libraries were generated using the BD Rhapsody Whole Transcriptome Analysis Amplification kit and sequenced on an Illumina Novaseq instrument. Raw sequencing reads were aligned to the GRCh38 human reference genome using the SevenBridges pipeline. Downstream analysis was processed using Seurat (v5.3.0) in R (v4.4.1). Raw count matrices were corrected for ambient RNA contamination using “decontX” from the celda package (v1.22.1), and downstream analysis was conducted on the corrected matrices. Cells were filtered based on the following quality control criteria: <200 or >7,000 detected genes per cell, >15% mitochondrial content, and >20% ribosomal content. Genes expressed in fewer than 10 cells and cells with high *MALAT1* expression were removed. To remove low-quality cells with abnormally high MALAT1 expression, we calculated the MALAT1 fraction per cell as the ratio of MALAT1 UMI counts to total UMI counts (MALAT1_fraction = MALAT1 UMIs/total UMIs) using the raw count matrix. Cells with a MALAT1_fraction ≥ 0.10 were excluded from downstream analyses. Following this filtering step, the MALAT1 gene itself was also removed from the feature list to prevent it from disproportionately influencing normalization and dimensionality reduction. Doublets were predicted and removed using “DoubletFinder” (expected doublet rate = 7.5%). Filtered count data were normalized and scaled with a scale factor of 10,000. Principal component analysis was performed based on the scaled data for the 3,000 highly variable genes identified.

### Integration and Cell-Type Annotation of Single-Cell RNA-seq Data

Integration across organoid samples was performed using Harmony on the 40 principal components. For downstream analysis, including construction of the k-nearest neighbor graph, clustering, and UMAP embedding, the top 30 Harmony-aligned dimensions were used. Clustering was performed using the Leiden algorithm at a resolution of 0.45. Clusters with fewer than 100 cells and those with abnormally high mitochondrial gene content were excluded as low-quality clusters. A total of 14 high-quality clusters were retained for subsequent analyses, except the cluster of mural cells.

Cell-type annotation was performed through a combination of label transfer and manual curation. Reference cell types were derived from a publicly available human fetal brain single-cell transcriptomic dataset (Bhaduri et al., 2020). The reference data were preprocessed by log-normalized counts, selecting 3,000 highly variable genes, and scaling the full matrix prior to PCA. Label transfer was carried out using “FindTransferAnchors” based on the PCA embeddings of the 30 dimensions. Cell identity labels were transferred to the organoid cells using “TransferData.”

Cluster marker genes were identified using “FindMarkers” with the Wilcoxon rank-sum test (min.pct=0.25, log2fold-change [FC] threshold=0.2). Significant cluster marker genes were defined as those with a log2FC > 1 at a false discovery rate (FDR) < 0.05. Predicted cell-type labels, top cluster marker genes, and canonical cell-type marker genes were collectively used to manually assign final cell-type identities.

### Differential Expression and Gene Ontology Analysis

For each cell type, differentially expressed genes (DEGs) were identified separately by comparing heterozygous or homozygous organoids to wild type using the “FindMarkers” with the MAST algorithm. Genes were considered significantly differentially expressed if expressed in at least 25% of cells (min.pct=0.25) and an absolute log2FC (|log2FC|) > log2(1.5) at FDR < 0.01. For downstream interpretation, over-representation analysis of DEGs was performed using the clusterProfiler package (v4.14.6). Upregulated and downregulated genes were analyzed separately for each cell type and comparison. Gene ontology (GO) enrichment analysis was conducted using “enrichGO” for biological processes, with gene set sizes restricted from 100 to 1,000 genes. Multiple testing correction was applied using the FDR adjustment, and significant GO terms were defined at FDR < 0.05. SynGO analysis was performed by GOAT (v1.1.2). Log2FC values were used to test for enrichment of SynGO gene sets (release version 20231201). Multiple testing correction was applied independently for each gene set source (SYNGO_CC, SYNGO_BP) using FDR adjustment, and terms with FDR < 0.05 were considered significant.

### Trajectory and Lineage Analysis Between Conditions

Pseudotime and cell trajectories were reconstructed to capture the dynamic progression of progenitor populations toward differentiated neuronal states. Cycling progenitors were designated as the root population, and Slingshot (v2.10.0) was applied to fit smooth principal curves through the UMAP embedding, thereby defining continuous developmental lineages. Each cell was assigned a pseudotime value, allowing quantitative representation of its transcriptional position along inferred trajectories. Lineage connectivity and trajectory structures were visualized in both 2-dimensional embeddings and schematic graphs to confirm consistency with expected neurodevelopmental hierarchies.

To assess whether *USP15* perturbation altered developmental trajectories, condiments (v1.4.0) was used to quantify local asymmetries in cell distribution between conditions and to reconstruct condition-specific trajectories based on a shared lineage skeleton. Pseudotime distributions were compared across wild-type, heterozygous, and homozygous mutant organoids. For each lineage, one-sided Wilcoxon rank-sum tests were conducted to assess directional shifts in pseudotime between all genotype pairs, and p-values were adjusted for multiple testing using the Benjamini-Hochberg method.

### Cell-Cycle and Potency Score Analysis

The cell-cycle score was quantified using curated Seurat gene sets representing the S, G2/M, and G1 phases of the cell cycle (CellCycleScoring()), generating per-cell continuous S and G2/M scores and a categorical phase assignment (G1, S, or G2/M). Cellular differentiation potency was quantified using CytoTRACE2 (v1.17) (Kang et al., 2025), which infers transcriptional diversity from stemness scores derived from single-cell RNA sequencing data. CytoTRACE2 was applied to the integrated dataset to generate continuous potency scores and categorical potency levels for all cells. These values were incorporated into downstream metadata and visualizations for comparison across conditions and cell types. Statistical evaluation of continuous score differences (S score, G2/M score, and CytoTRACE2 potency) between genotypes was performed within each cell type using 2-sided Wilcoxon rank-sum tests with the Benjamini-Hochberg correction for multiple testing.

### Cellular Stress Program Module Score Analysis

To characterize apoptotic, senescent, and proteotoxic stress states in progenitor and neuronal populations, 8 gene-program modules, including Pro-apoptosis, Anti-apoptosis, Senescence, senescence-associated secretory phenotype (SASP), ER stress, DNA damage, Oxidative stress, and Heat shock, were scored per cell using scanpy.tl.score_genes (Scanpy v1.10) on log-normalized counts with default control gene set parameters. Module gene lists were curated from KEGG pathways and complementary public gene set resources, as follows: Pro-apoptosis and Anti-apoptosis modules from KEGG hsa04210 (Apoptosis), partitioned into BCL-2-family pro- versus anti-apoptotic effectors according to (Singh et al., 2019); Senescence from KEGG hsa04218 (Cellular senescence); SASP from KEGG hsa04218 combined with hsa04668 (TNF signaling pathway); ER stress from KEGG hsa04141 (Protein processing in the endoplasmic reticulum); DNA damage from KEGG hsa03440 (Homologous recombination) combined with hsa04115 (p53 signaling pathway); Oxidative stress from KEGG hsa05208 (Chemical carcinogenesis—reactive oxygen species) combined with the MSigDB v2023.2 gene set GO:0006979; and Heat shock from the HSF1/HSP gene subset of KEGG hsa04141, with additional inclusion of HSPB1 and HSPB8. Within each cell population (Pooled, 7 progenitor subtypes, and 7 neuronal subtypes), mutant versus WT module score comparisons were performed for each module using the 2-sided Mann-Whitney *U*-test, and *P* values were adjusted across all tests using the Benjamini-Hochberg method (FDR < 0.05).

### Inference of Gene Regulatory Networks and Enriched Signaling Pathways

Transcription factor (TF) regulatory network analysis was performed using the pySCENIC (v0.12.1) workflow (Van de Sande et al., 2020). For the gene regulatory network (GRN) inference step, the RegDiffusion algorithm was used instead of the default GRNBoost2 (Zhu and Slonim, 2024). RegDiffusion applies a diffusion-based sparse regression approach to identify nonlinear co-expression relationships between TFs and candidate target genes, generating an adjacency matrix of TF-target gene pairs with importance scores. A curated list of 1639 human TFs from the cisTarget resource was used to infer GRNs. For each gene, the top 20 regulators (*k* = 20) were retained, and edges with importance scores below the 50th percentile were filtered.

The inferred TF-target interactions were refined using the cisTarget motif ranking database (hg38, version 10), retaining only those supported by motif enrichment. The biological activity of each regulon was quantified at the single-cell level using AUCell, evaluating whether regulon genes are enriched at the top of each cell's ranked expression profile. To compare regulon activity between conditions, we summarized regulon activity scores by computing the median per cell type and performed statistical testing using the Wilcoxon rank-sum test. Multiple testing was adjusted using the Benjamini-Hochberg method, and statistical significance was defined as FDR < 0.05.

Heatmaps were generated to compare WT vs *USP15* Het and WT vs *USP15* Hom samples, visualizing only TFs with significant median differences in regulon activity. For network analysis, downregulated TFs were mapped to downregulated DEGs based on inferred TF-target interactions, restricted to associations consistently observed in at least 5 cell types for the *USP15* heterozygous condition and 7 cell types for the *USP15* homozygous mutant condition.

For TF expression-regulon comparison, the gene expression levels of SOX2, NR2F1, and NR2F2 and the AUCell scores of their corresponding regulons were summarized within each cell population and compared between USP15 Het and WT organoids. To assess the relationship between progenitor TF regulon activity and stress-associated transcriptional programs, partial Spearman correlations were computed between SOX2, NR2F1, or NR2F2 regulon AUCell scores and ER stress, oxidative stress, heat shock, or DNA damage module scores within each genotype and cell population. S-phase score, G2/M score, and detection rate were included as covariates. P-values were adjusted using the Benjamini-Hochberg method, and FDR < 0.05 was considered significant.

### HOX Regulon Activity and Regional Identity Analyses

HOX-focused analyses were performed on Hom progenitor populations. Per-cell HOX activity was calculated as the mean AUCell score across HOX-family regulons that were upregulated in Hom compared with WT. Regional identity scores were computed using the region-specific gene sets for the developing human brain (Braun et al., 2023). HOX-family genes were excluded from posterior gene sets to avoid circularity. The anterior-posterior score was defined as the difference between the anterior and posterior regional scores. Associations between HOX activity and anterior-posterior scores were tested within each progenitor subtype using Spearman correlation with Benjamini-Hochberg correction.

Anterior identity scores were computed from telencephalon and forebrain gene sets. Associations between HOX activity and anterior identity scores were assessed with partial Spearman correlation, controlling for S-phase score, G2/M score, and detection rate (nFeature_RNA divided by the maximum nFeature_RNA value). HOX-high and HOX-low Hom progenitors were defined by the upper and lower tertiles of per-cell HOX activity, excluding cells in the middle tertile. Differential expression between HOX-high and HOX-low Hom progenitors was performed using MAST with the same thresholds used for cell-type-specific DEG analysis.

HOX-positive cells were defined by detectable expression of HOX-family genes. Pairwise co-expression similarity among HOX paralog groups was computed using Jaccard similarity, and its association with paralog-group distance was assessed with Spearman correlation. Stress module scores for ER stress, oxidative stress, heat shock, and DNA damage programs were computed for each cell. Associations between HOX activity and stress module scores were evaluated with partial Spearman correlation, controlling for S-phase score, G2/M score, and detection rate. Multiple testing correction was performed with the Benjamini-Hochberg method, and adjusted p-values below 0.05 were considered significant.

### Disease Risk Genes and Gene Set Enrichment Analysis

We compiled comprehensive sets of risk genes implicated in neurodevelopmental and neuropsychiatric disorders from large-scale genomic studies. For ASD, we included 72 and 185 ASD risk genes identified by the transmission and de novo association (TADA) framework in Fu et al. (2022) (ASD72, TADA FDR ≤0.001; ASD185, FDR ≤0.05). Additionally, we identified 102 genes associated with ASD, as reported by Satterstrom et al (2020) (Satterstrom102, FDR ≤0.1). For developmental disorder (DD), 309 DD and 477 genes were obtained from Fu et al. (2022) (DD309, TADA FDR ≤0.001; DD477, TADA FDR ≤0.05). For neurodevelopmental disorder (NDD), 373 NDD risk genes and 664 NDD risk genes were obtained from Fu et al. (2022) (NDD373, TADA FDR ≤0.001; NDD664, TADA FDR ≤0.05). For schizophrenia (SCZ), 10 and 224 risk genes were obtained from Singh et al. (2022) (SCZ10, *P*<2.14x10^−6^; SCZ224, *P*< .01). These curated gene sets were used in enrichment tests on significant cell-type-specific DEGs identified between wild-type and *USP15* mutant organoids. All enrichment analyses were performed using one-sided Fisher’s exact tests with correction for multiple comparisons by Benjamini-Hochberg.

### Differentially Expressed Genes Analysis of Genetic Perturbation Effects in the Mouse Brain Cell Atlas With Perturbations

The mouse brain cell atlas with perturbations of ASD-associated genes was obtained from a published dataset on Zenodo (https://doi.org/10.5281/zenodo.17112344). To identify gene expression changes associated with cell-type-specific gene perturbations in the mouse brain cell atlas, we performed differential expression analysis using a pseudobulk framework. Raw counts were then aggregated into sample-level pseudobulk profiles by pairing this identifier with cell type. Aggregation was performed using decoupler-py (v1.9.2) (Badia et al., 2022), with samples retained only if they contained more than 10 cells and more than 1,000 total counts per cell type. For differential expression analysis, we performed within-dataset pairwise comparisons between perturbed and normal pseudobulk samples from the same dataset, requiring at least 2 samples per group and a minimum of 100 pseudobulk cells per group. Pseudobulk count matrices were analyzed for differential expression testing using edgeR (v4.4.2) (Chen et al., 2025). Genes with less than 10 counts were removed, and normalization factors were computed. Differential expression was assessed using the quasi-likelihood framework. Genes were considered significantly differentially expressed if they passed a nominal *P* value less than 0.05 and an absolute log2-transformed fold change greater than 0.1. We then evaluated the overlap between cell-type-specific DEGs of *USP15* mutant organoids and those obtained from mouse brain cells with perturbations. Enrichment of *USP15*-associated DEGs within each mouse perturbation DEGs was assessed using one-sided Fisher’s exact tests, with multiple testing correction applied by the Benjamini-Hochberg method.

## RESULTS

### Generation of USP15 Mutant iPSCs and Brain Organoids

In our previous study (Kim et al., 2024a), we identified a de novo start-codon-disrupting variant in *USP15* (GRCh38: chr12:62,260,415 A>T; ENST00000280377.10: c.1A>T; p.Met1Leu) in an individual with ASD (IBS-ASD-12693). The patient exhibited core ASD symptoms with an ADOS-2 total score of 5, ADI-R scores of 24 for social interaction, 19 for communication, 9 for repetitive behaviors, and SCQ scores of 8 for current and 20 for lifetime. Within the Korean ASD cohort, these scores showed no significant deviations across clinical domains, although the patient showed a trend toward elevated repetitive and restricted behaviors (ADI-R domain C; z-score = 1.51). This variant was predicted to be damaging by multiple computational tools, with a CADD Phred score of 25.4, a missense constraint z-score of 3.50, and a JARVIS score greater than 0.99, consistent with a potentially deleterious effect of the variant.

In the developing human brain, genes involved in the ubiquitin system components, such as *UBB*, *UBC*, and *USP34*, exhibit robust expression across multiple cell types from early progenitor stages (Kim et al., 2024b) (Supplementary Fig. 1A). Consistent with this pattern, *USP15* is broadly expressed across various cell types during human brain development, including neural progenitors such as radial glia and neuroblasts (Supplementary Fig. 1B). *USP15* expression begins as early as gestational week 5 in radial glia cells and neuroblasts, peaks in mid-fetal neuronal populations, and remains consistently high across neuronal cell types during development (Supplementary Fig. 1B). In addition, *USP15* shows strong haploinsufficiency as shown in the gnomAD pLI database (pLI score = 1). This pattern is common among high-confidence ASD risk genes (Fu et al., 2022, Kim et al., 2025), with 156 of 185 ASD-associated genes showing haploinsufficiency (pLI > 0.9). Similarly, a substantial proportion of other ubiquitin-related genes are also haploinsufficient (pLI > 0.9; 26 out of 79 genes).

We thus engineered isogenic hiPSC lines carrying the USP15 p.Met1Leu allele in heterozygous (Het) and homozygous (Hom) states using CRISPR/Cas9. On-target edits were verified by Sanger sequencing (Supplementary Fig. 2A), and USP15 protein levels were reduced in Het and absent in Hom by immunoblot, consistent with loss of function (Supplementary Fig. 2B). No sequence alterations were detected at the 6 top in silico-predicted off-target loci (≤3 mismatches) (Supplementary Fig. 3).

**Fig. 1:**
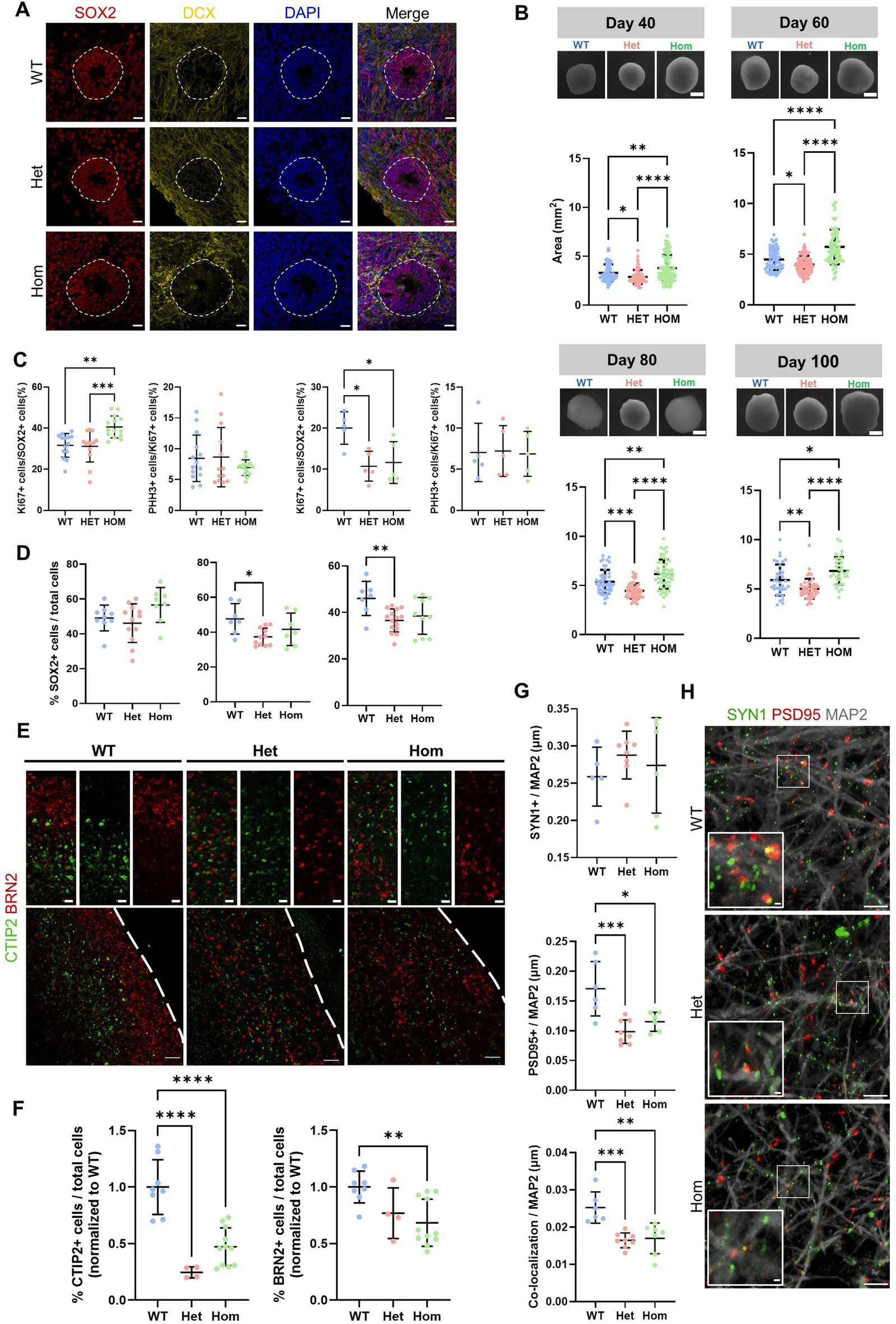
Generation and phenotypic characterization of USP15 mutant brain organoids. **(A)** Representative immunostaining for SOX2 (red), DCX (yellow), and DAPI (blue) in wild-type (WT), USP15 heterozygous (Het), and USP15 homozygous (Hom) brain organoids at day 40. Scale bar, 20 μm. **(B)** Representative bright-field images and quantification of cross-sectional area of WT, Het, and Hom brain organoids at days 40, 60, 80, and 100. Scale bar, 1 mm. Each dot represents the raw area measurement of an individual organoid. Organoids were analyzed from independent differentiation batches as follows: day 40, 5 batches, WT *n* = 79, Het *n* = 71, Hom *n* = 99; day 60, 5 batches, WT *n* = 66, Het *n* = 94, Hom *n* = 78; day 80, 4 batches, WT *n* = 47, Het *n* = 56, Hom *n* = 54; day 100, 4 batches, WT *n* = 35, Het *n* = 47, Hom *n* = 35. Data are presented as mean ± SD. For each time point, statistical significance was assessed using a linear model including genotype and differentiation batch as covariates. **(C)** Quantification of Ki67-positive cells among SOX2-positive progenitor cells and PHH3-positive cells among Ki67-positive cells in WT, Het, and Hom organoids at days 30 and 100. Organoids were analyzed from independent differentiation batches as follows: day 30, 3 batches, WT *n* = 14, Het *n* = 13, Hom *n* = 14; day 100, 1 batch, WT *n* = 5, Het *n* = 5, Hom *n* = 5. Each dot represents one organoid. **(D)** Quantification of the percentage of SOX2-positive neural progenitor cells among total DAPI-positive cells in WT, Het, and Hom organoids at days 60, 80, and 100. Organoids were analyzed from 2 independent differentiation batches: day 60, WT *n* = 9, Het *n* = 12, Hom *n* = 8; day 80, WT *n* = 7, Het *n* = 11, Hom *n* = 7; day 100, WT *n* = 7, Het *n* = 15, Hom *n* = 10. Each dot represents one organoid. **(E)** Representative immunostaining for CTIP2 (green) and BRN2 (red) in WT, Het, and Hom organoids at day 100. Upper panels show higher-magnification images of CTIP2-/BRN2-positive cells, and lower panels show wider views of the cortical-like neuronal region. Dashed lines mark the organoid boundary. Scale bars, 20 μm in upper panels and 50 μm in lower panels. **(F)** Quantification of the fractions of CTIP2-positive and BRN2-positive cells among total DAPI-positive cells in day 100 WT, Het, and Hom organoids. Values were normalized to WT. Organoids were analyzed from 2 independent differentiation batches: WT *n* = 8, Het *n* = 4, Hom *n* = 11. Each dot represents one organoid. **(G)** Quantification of SYN1 puncta, PSD95 puncta, and SYN1-PSD95 colocalized puncta normalized to MAP2-positive dendrite length in day 100 WT, Het, and Hom organoids. Synaptic puncta were quantified from selected MAP2-positive neuronal layer ROIs. Day 100 organoids from 2 independent differentiation batches were analyzed: WT *n* = 6, Het *n* = 8, Hom *n* = 7. For each organoid, 4 or more ROIs were analyzed and averaged to generate one organoid-level value. Each dot represents one organoid. **(H)** Representative immunostaining for MAP2 (dark gray), SYN1 (green), and PSD95 (red) in WT, Het, and Hom organoids at day 100. Images were acquired from 38.48- × 38.48-μm MAP2-positive neuronal layer ROIs using identical confocal acquisition settings across genotypes. Boxed regions show higher-magnification views highlighting SYN1 and PSD95 puncta. Scale bar, 5 μm; inset box, 1 μm. For panels C, D, F, and G, data are presented as mean ± SD and statistical significance was assessed by one-way ANOVA unless otherwise indicated. Statistical significance is indicated as *P < .05, **P < .01, ***P < .001, and ****P < .0001.

Unguided brain organoids were generated from isogenic wild-type (WT), Het, and Hom hiPSC lines using a published protocol and maintained to day 107 (Giandomenico et al., 2021). At day 40, all genotypes exhibited neuroepithelial rosettes with SOX2^+^ ventricular zone-like epithelia and emerging DCX^+^ immature neurons (Fig. 1A). Growth was monitored by measuring the organoid cross-sectional area at days 40, 60, 80, and 100. After accounting for batch, model-based estimates showed Het consistently smaller and Hom larger than WT at each time point (Fig. 1B). To further assess progenitor proliferation and mitotic progression, we quantified Ki67-positive cells among SOX2-positive progenitors and PHH3-positive cells among Ki67-positive cells at days 30 and 100. Hom organoids showed an increased Ki67/SOX2 ratio at day 30 but a reduced Ki67/SOX2 ratio at day 100, whereas the PHH3/Ki67 ratio did not show significant genotype-dependent differences at either time point (Fig. 1C). Progenitor abundance (SOX2^+^/DAPI^+^) was comparable among genotypes at day 60 (ANOVA, *P* = .08) but diverged thereafter: Het was lower than WT at day 80 (∼22% lower) and day 100 (∼21% lower; Tukey-adjusted *P* < .05), whereas Hom did not differ from WT at either time point (Fig. 1D). At day 100, we examined CTIP2 (BCL11B) and BRN2 (POU3F2) as deep- and upper-layer markers; representative images exhibited a less radially ordered appearance of CTIP2^+^ labeling in Het and Hom relative to WT (Fig. 1E). Quantification of CTIP2^+^/DAPI^+^ showed lower fractions in both Het and Hom relative to WT, whereas BRN2^+^/DAPI^+^ fractions were comparable to WT in Het but reduced in Hom (Fig. 1F). For synaptic readouts at day 100, SYN1 (presynaptic) and PSD95 (postsynaptic) puncta were quantified per unit MAP2-positive dendrite length; PSD95 puncta density and SYN1-PSD95 colocalized puncta were reduced in both Het and Hom relative to WT, whereas SYN1 puncta were comparable to WT (Fig. 1G, H).

### A Single-Cell Transcriptomic Analysis Reveals Genotype-Dependent and Progenitor-Centric Dysregulation in *USP15* Mutant Organoids

**Fig. 2:**
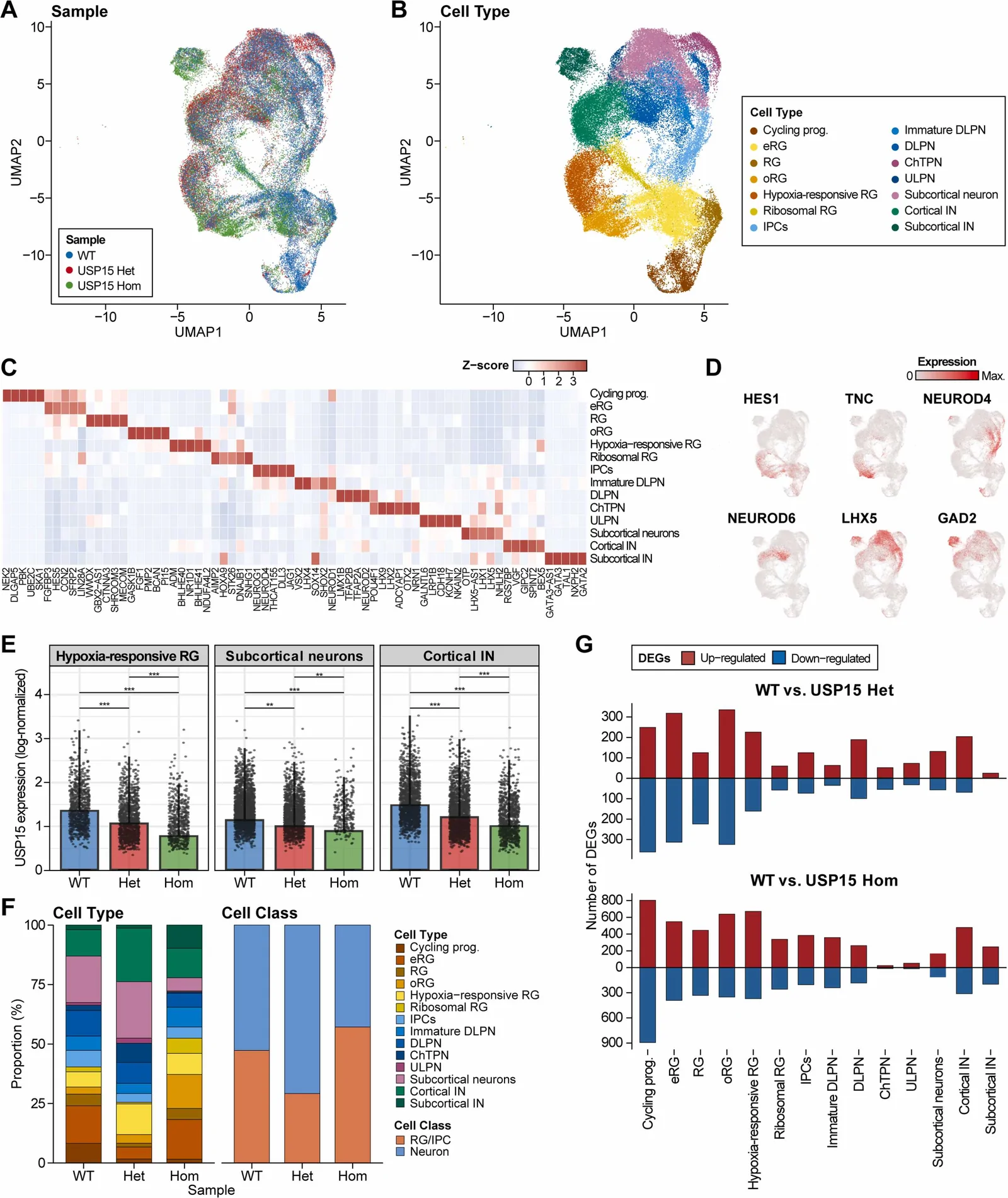
*Single-cell transcriptomic atlas of WT and USP15 mutant brain organoids.***(A)** UMAP of all high-quality cells from day 107 organoids after integration, colored by genotype (WT, Het, and Hom). **(B)** UMAP of 14 cell types. Cycling prog., cycling progenitors; eRG, early radial glia; RG, radial glia; oRG, outer radial glia; Hypoxia-responsive RG, hypoxia-responsive radial glia; Ribosomal RG, ribosomal radial glia; IPCs, intermediate progenitor cells; Immature DLPN, immature deep-layer projection neurons; DLPN, deep-layer projection neurons; CThPN, corticothalamic projection neurons; ULPN, upper-layer projection neurons; Cortical IN, cortical interneurons; Subcortical IN, subcortical interneurons **(C)** Heatmap of top 10 cell-type-specific marker genes across cell types. **(D)** UMAP of gene expression of canonical markers. **(E)***USP15* expression (log-normalized) within hypoxia-responsive radial glia, cortical interneuron, and subcortical neuron clusters by genotype. Boxes show medians; points are single cells. **: FDR < 0.01; ***: FDR < 0.001 by Wilcoxon test. **(F)** Composition of 14 cell types and cell classes per sample. **(G)** Number of differentially expressed genes (DEGs) per cell type comparing Het versus WT (top) and Hom versus WT (bottom).

To elucidate genotype-dependent consequences of *USP15* insufficiency, we performed single-cell RNA sequencing (scRNA-seq) on WT, Het, and Hom organoids at day 107. Across genotypes, 62,897 high-quality cells were retained after quality control and integrated, yielding 14 transcriptionally distinct populations (Fig. 2A, B, Supplementary Fig. 4A, Supplementary Table 1). Using a human developing cortex atlas (Bhaduri et al., 2020) and canonical marker genes (Bhaduri et al., 2020, Hendriks et al., 2024, Uzquiano et al., 2022), we annotated major cell populations expected during early neurodevelopment and identified marker genes for each cell type (Fig. 2C, D, Supplementary Fig. 4B-D, Supplementary Table 2A). These included diverse progenitor populations, such as cycling progenitors, early radial glia (eRG), outer radial glia (oRG), and intermediate progenitors (IPC), as well as neuronal subtypes, including cortical interneurons, subcortical neurons, and deep- and upper-layer projection neurons (Fig. 2B, C).

*USP15* expression levels corresponded with the genotype, showing higher expression in WT and progressively reduced levels in Het and Hom across multiple progenitor and neuronal populations. In early RG, both Het (log_2_FC = −0.36, FDR = 4.42x10^−5^) and Hom (log_2_FC = −0.80, FDR = 2.60x10^−23^) showed significant downregulation compared with WT (Supplementary Table 2B). As representative examples, significant stepwise decreases were also observed in hypoxia-responsive radial glia, subcortical neurons, and cortical interneurons, with Het exhibiting an intermediate level between those of WT and Hom (Fig. 2E**,** Supplementary Table 2C).

Consistently, we observed a significant reduction in USP15 mRNA levels in both Het and Hom mutant lines compared with the WT in RT-qPCR analysis (Supplementary Fig. 2C). This marked decrease in mRNA levels, despite the start-codon-disrupting mutation, is highly suggestive of translation-mediated mRNA decay. Previous studies have established that constraints or delays in translation initiation can actively trigger mRNA destabilization and decay pathways (eg, No-Go Decay) (Gaither et al., 2021, Kozak, 1989, Powers et al., 2020, Presnyak et al., 2015, Simms and Yan, 2017).

Analysis of cellular composition revealed distinct genotype-dependent shifts in population abundances (Fig. 2F, Supplementary Table 2D). Consistent with histological observations (Fig. 1D), in Het, progenitor subtypes accounted for a smaller proportion of total cells compared with WT (WT: 47.32%, Het: 29.21%), consistent with reduced representation of the progenitor pool. In Hom, the overall progenitor fraction was similar to WT, but the composition shifted, with fewer cycling progenitors (WT: 8.25%, Hom: 1.57%) and more oRG (WT: 3.03%, Hom: 14.28%). Hom also showed a marked reduction in subcortical neurons (WT: 19.55%, Het: 23.72%, and Hom: 5.53%). These consistent genotype-specific patterns suggest distinct genotype-associated effects on progenitor and neuronal cell populations.

To assess which cell types were most affected by *USP15* loss, we performed differential expression analysis across annotated cell populations, comparing WT and mutant samples. The majority of DEGs were concentrated in the progenitor populations (Fig. 2G, Supplementary Tables 2E and F). In the Het, progenitor subtypes such as eRG (629 DEGs) and oRG (657 DEGs) each exhibited over 600 significant DEGs (Supplementary Table 2E). In contrast, deep-layer projection neurons, the most transcriptionally affected neuronal population, showed 286 DEGs, fewer than half the number observed in eRG or oRG. In the Hom, a greater number of significant DEGs were observed across overall cell types compared with Het (Supplementary Table 2F). Progenitor subtypes such as eRG (934 DEGs) and oRG (984 DEGs) each exhibited more than 900 DEGs. Neuronal cell types consistently showed fewer DEGs than progenitor cells. Among neuronal cell types, immature deep-layer projection neurons, the neuronal cell type with the most DEGs, showed 593 DEGs, suggesting that neuronal cells were less affected than progenitor cells. Hom showed an even more pronounced progenitor bias, suggesting more extensive transcriptional alterations in progenitor populations in the homozygous loss state.

### *USP15* Genotypes Differentially Alter Progenitor Gene Programs and Developmental Trajectories

**Fig. 3:**
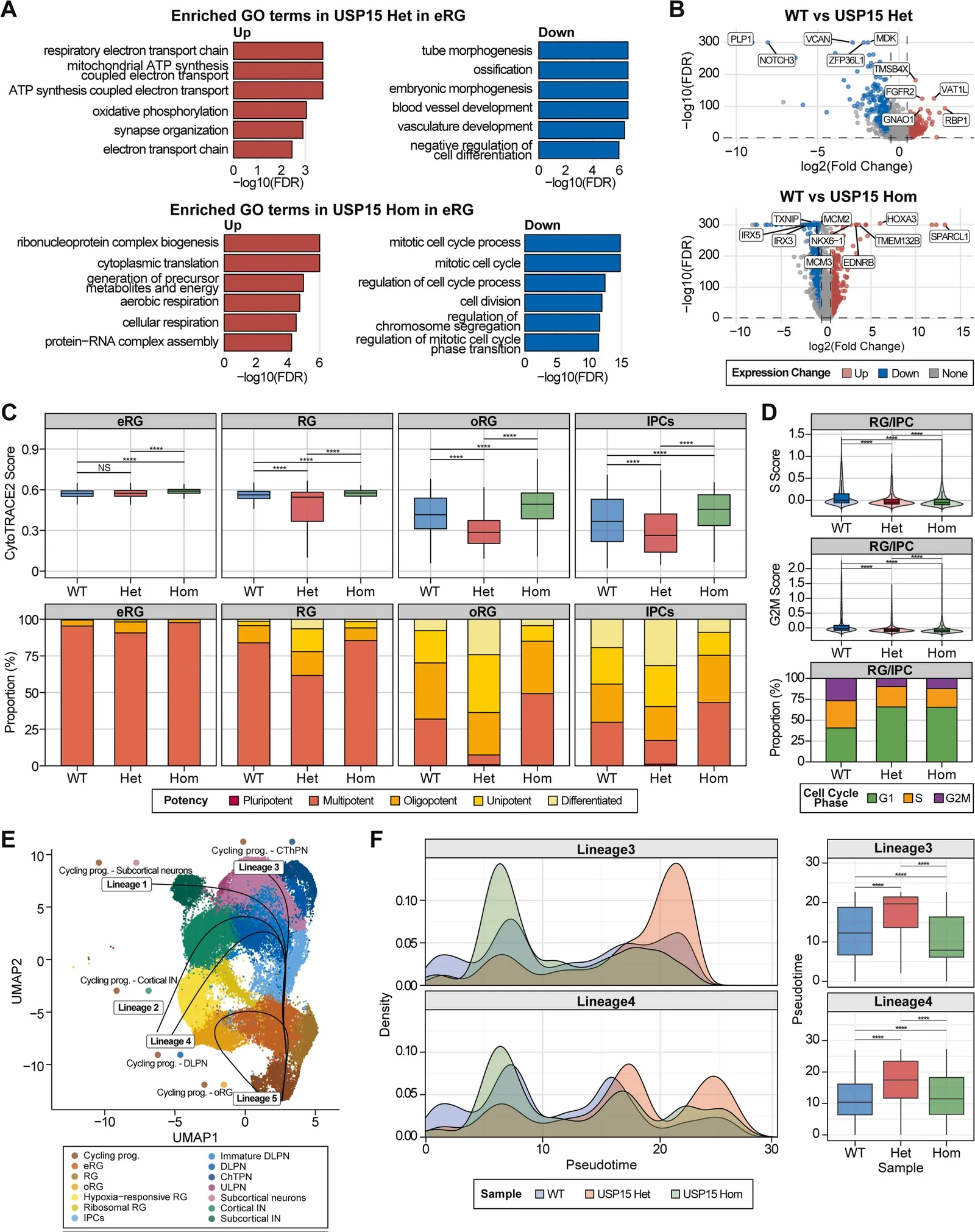
*Progenitor transcriptional programs, cell-state metrics, and developmental trajectories altered in USP15 mutants.***(A)** Gene Ontology (GO) enrichment analysis of DEGs in eRG (FDR < 0.05). **(B)** Volcano plots of DEGs in eRG comparing WT and *USP15* mutants. **(C)** Comparison of potency score (top) and stages (bottom) between WT and *USP15* mutants across progenitor cell subtypes. Significance levels are based on adjusted *P* values by FDR from pairwise Wilcoxon tests. ns: not significant, *: FDR < 0.05, **: FDR < 0.01, ***: FDR < 0.001, ****: FDR < 0.0001. **(D)** Cell-cycle distribution in all radial glial subtypes and intermediate progenitor cell populations. Comparison of G2M scores (top) and phases (bottom) between WT and *USP15* mutant. Significance levels are based on adjusted p-values by FDR from pairwise Wilcoxon tests. ns: not significant, *: FDR < 0.05, **: FDR < 0.01, ***: FDR < 0.001, ****: FDR < 0.0001. **(E)** Trajectory lineage inference with Slingshot for 5 major lineages. **(F)** Pseudotime distributions of WT and *USP15* mutants represented by a density plot (left) and boxplots (right) along lineages 3 and 4. Significance levels are based on adjusted *p* values by FDR from pairwise Wilcoxon tests. ns: not significant, *: FDR < 0.05, **: FDR < 0.01, ***: FDR < 0.001, ****: FDR < 0.0001.

To examine genotype-dependent perturbations of progenitor programs, we focused on eRG and cycling progenitors, as these proximal progenitor states exhibited particularly high DEG burdens. In Het, eRG showed reduced expression of genes that support progenitor maintenance and the periventricular niche (*NOTCH3, ZFP36L1*, and ECM/vascular factors) (Fig. 3A, B, Supplementary Table 2E, 3A). Downregulated GO terms included negative regulation of cell differentiation, tube and embryonic morphogenesis, and vasculature development. In contrast, upregulated genes were enriched in oxidative phosphorylation, respiratory electron transport, and synapse organization. These changes reflected a loss of stemness-associated modules, accompanied by increased bioenergetic and neuron-associated programs, consistent with neurogenic priming and a shift toward cell-cycle exit.

In Hom, eRG showed broad suppression of mitotic programs. Key mitotic regulators (*CDK1*, *MKI67*, and *TOP2A*) were strongly downregulated, with enrichment for GO terms related to the mitotic cell cycle and chromosome segregation. Upregulated genes were predominantly associated with biosynthetic and metabolic pathways, with enrichment for ribonucleoprotein complex biogenesis and aerobic respiration, suggesting that altered metabolic demands accompany cell-cycle suppression (Fig. 3A, B, Supplementary Table 2F, 3B). In addition, focal activation of several HOX-family genes (*HOXA7/9, HOXB2/3*) and the ECM-related gene *SPARCL1* was observed, suggesting broadly aberrant activation of HOX-family genes in Hom eRG (Fig. 3B).

Cell-state metrics for potency and cell cycle distinguished the 2 genotypes across multiple progenitor populations (Fig. 3C, D, Supplementary Fig. 5A, Supplementary Table 3C). Progenitors of Het exhibited lower CytoTRACE2 scores, consistent with earlier maturation and a reduced proliferative state, with a reduced G2/M phase and relative G1 enrichment, consistent with early cell-cycle exit. By contrast, Hom showed higher developmental potency scores overall than WT and exhibited distinct cell-cycle features, indicating retention of a progenitor-like transcriptional state despite altered cell-cycle progression. In pooled Hom progenitors, the G1 fraction was increased relative to WT, with corresponding reductions in S and G2/M fractions and module scores (Fig. 3D). Across progenitor subtypes, this phase redistribution was most pronounced in oRG and IPCs, where the G1 fraction increased from 73.4% to 88.1% in oRG and from 64.8% to 90.4% in IPCs, whereas eRG and RG showed comparable phase proportions between WT and Hom (Supplementary Fig. 5A, Supplementary Table 3C). S-phase and G2/M module scores were significantly reduced in Hom progenitors compared with WT across all progenitor subtypes, including eRG, RG, oRG, and IPCs (FDR < 0.05, Mann-Whitney U-test) (Supplementary Fig. 5A, Supplementary Table 3C). To determine whether this phenotype reflected apoptotic cell loss, we performed cleaved caspase-3 immunostaining at days 30 and 100, which did not reveal significant genotype-dependent differences in apoptotic cells (Supplementary Fig. 5B). In parallel, cellular stress program scores showed significant increases in senescence and oxidative stress programs across Hom progenitor subtypes, along with reduced or nonelevated SASP scores in progenitor subtypes and increased anti-apoptotic programs in RG, oRG, and IPCs (FDR < 0.05, Mann-Whitney *U*-test) (Supplementary Fig. 5C, Supplementary Table 3D). The Hom progenitor phenotype was accompanied by stress- and survival-associated transcriptional changes rather than broad activation of apoptotic programs.

To place transcriptional and cell-differentiation alterations of progenitors in a developmental context, we inferred that 5 differentiation-trajectory lineages emerged from cycling progenitors and extended toward distinct endpoints (Fig. 3E**,** Supplementary Fig. 6A, B). Among lineages, lineages 3 and 4 corresponded to cortical projection neuronal differentiation trajectories. Lineage 3 was directed toward corticothalamic projection neurons (WT: 16,439 cells, Het: 6,913 cells, and Hom: 6,835 cells), while lineage 4 was directed toward deep-layer projection neurons (WT: 14,914 cells, Het: 5,838 cells, and Hom: 8,520 cells). Imbalanced scores of lineage assignment between samples were close to zero across genotypes, and no genotype-specific lineages emerged (Supplementary Fig. 6C, D). Instead, genotype-associated differences appeared in the distribution of cells along these existing lineages.

Comparing pseudotime for neuronal lineages across genotypes, we observed lineage-specific shifts in differentiation dynamics (Fig. 3F, Supplementary Table 3E). In lineage 3, extending from cycling progenitors toward corticothalamic projection neurons, Het organoid showed a shift toward later pseudotime states relative to WT (median pseudotime of WT = 12.1, Het = 19.5; FDR = 2.2x10^−16^, one-sided Wilcoxon rank-sum test), whereas Hom organoid showed a shift toward earlier pseudotime states (median pseudotime of Hom = 7.69; FDR = 1.35x10^−73^) (Fig. 3F). In lineage 4, progressing toward deep-layer projection neurons, Het organoid showed a shift toward later pseudotime dynamics relative to WT (median pseudotime of WT = 9.96, Het = 13.9; FDR = 2.2x10^−16^), and Hom organoid also showed a statistically significant difference in pseudotime distribution relative to WT (median pseudotime of Hom = 9.94; FDR = 8.26x10^−42^). These results suggest that *USP15* mutant genotypes are associated with lineage-dependent differences in progenitor-to-neuron trajectories, with haploinsufficiency associated with later pseudotime states and homozygous loss of *USP15* resulting in variable alterations across trajectories.

### Genotype-Dependent Changes in Neuronal Maturation and Synaptogenic Programs

**Fig. 4:**
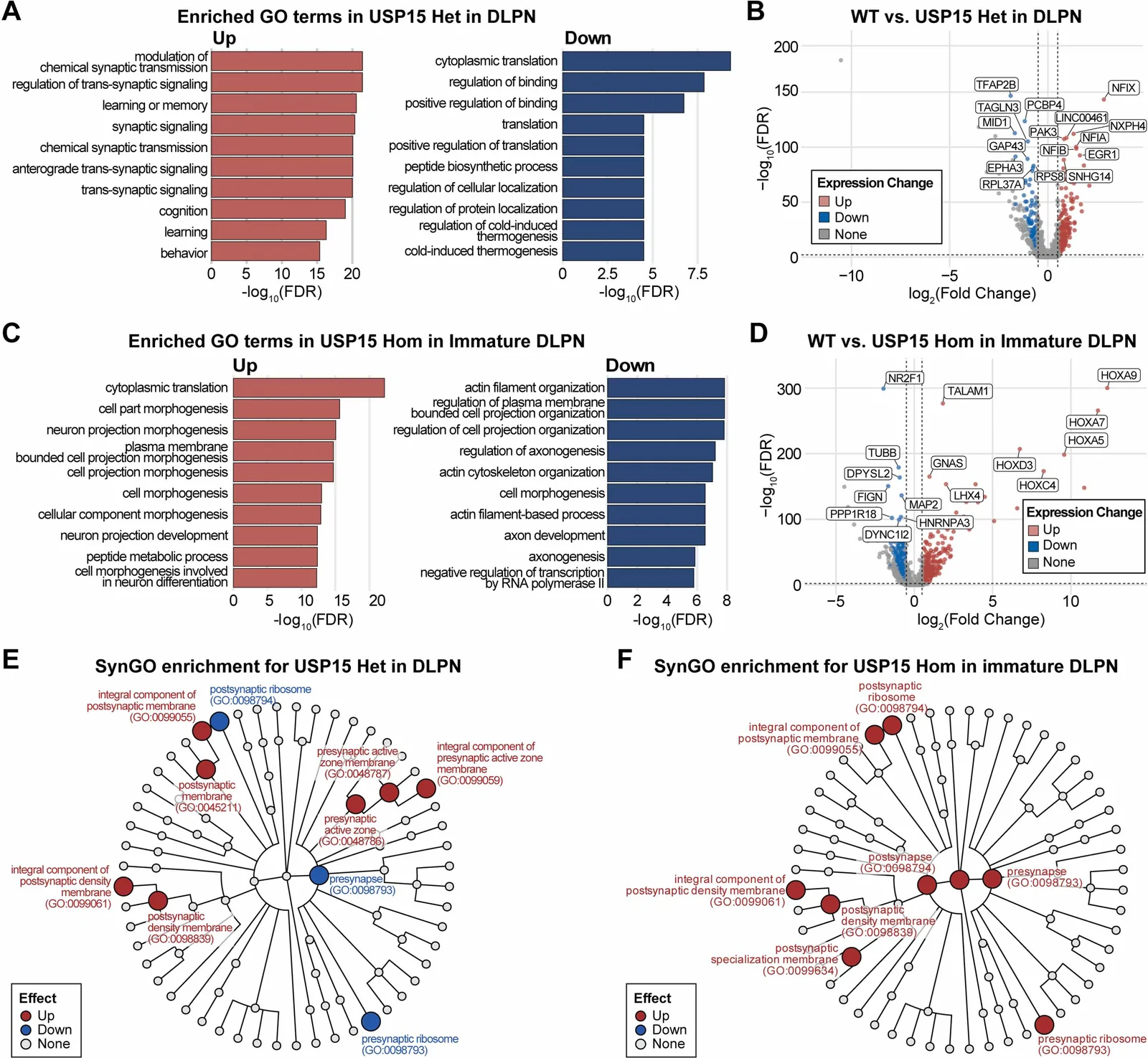
*Altered biological processes and synaptic components in USP15 mutant cortical projection neurons.***(A)** GO enrichment for significant DEGs of *USP15* Het in deep-layer projection neurons. **(B)** Volcano plot of DEGs of *USP15* Het in deep-layer projection neurons. **(C)** GO enrichment for significant DEGs of *USP15* Hom in immature deep-layer projection neurons. **(D)** Volcano plot of DEGs of *USP15* Hom in immature deep-layer projection neurons. **(E)** SynGO result of cellular component for *USP15* Het in deep-layer projection neurons. **(F)** SynGO result of cellular component for *USP15* Hom in immature deep-layer projection neurons.

Neuronal populations with a high number of DEGs showed marked alterations in maturation and synaptic programs of projection neurons, particularly deep-layer projection neurons in Het and immature projection neurons in Hom (Fig. 2G). In deep-layer projection neurons of Het, upregulated genes were significantly enriched for GO terms related to synaptic and trans-synaptic signaling (Fig. 4A, B**,** Supplementary Table 3A). Top DEGs included the trans-synaptic neurexin ligand *NXPH4* and the postsynaptic regulator *PAK3*, as well as other key postsynaptic receptors (*NTRK2*, *GRIN2B*, and *GRIA2*). Downregulated genes were associated with translation, protein binding, and cellular localization (Fig. 4A, Supplementary Table 3A).

To investigate these synaptic features further, we performed SynGO analysis, which revealed bidirectional changes in presynaptic categories together with enrichment of postsynaptic categories (Fig. 4E, Supplementary Table 4A). Within the presynaptic categories, upregulated genes were associated with active-zone organization, vesicle release, trans-synaptic adhesion, and receptor-linked signaling, whereas downregulated genes were enriched for presynaptic ribosome, local translation, axonal growth, calcium channel, and vesicle-trafficking programs. Consistent with this imbalance, *GAP43*, a presynaptic growth and axon guidance factor, was among the top downregulated genes (Fig. 4B). Together, these findings suggest early activation of postsynaptic programs accompanied by reduced presynaptic maturation-related programs and local translational support, consistent with the decreased *SYN1-PSD95* colocalization observed at day 100 (Fig. 1G). *DLG4*, which encodes PSD95, and *SYN1* were not significantly altered in Het deep-layer projection neurons, despite broader upregulation of curated synaptic genes across postsynaptic scaffold (*DLG2*, *SHANK1*, *SHANK2*, *SYNGAP1*, and *HOMER1*), presynaptic vesicle/release-related (*SYP*, *SYT1*, and *VAMP2*), and adhesion/receptor categories (*NRXN1*, *GRIN2B*, and *GRIA2*) (Supplementary Table 4B). Thus, the Het synaptic phenotype is unlikely to reflect reduced transcription of *DLG4* or *SYN1* alone. Rather, this result suggests partial uncoupling between synaptic transcriptional activation and puncta-level synaptic organization.

In immature deep-layer projection neurons of Hom, we observed more pronounced dysregulation. Top upregulated DEGs were enriched for HOX-family genes (*HOXA7*, *HOXB2*, and *HOXB3*), suggesting aberrant HOX-family activation (Fig. 4C, D, Supplementary Table 3B). Downregulated GO terms and DEGs indicated reduced axonogenesis, cytoskeletal organization, and cell-projection development, including *MAP2*, *EPHA4*, and *SYNGAP1*, consistent with impaired structural maturation of neural processes (Fig. 4C, D, Supplementary Table 3B). Pre- and postsynaptic categories were enriched in SynGO, but the signal was led by translational modules and cytoskeletal and endocytic regulators rather than core presynaptic release or active zone components (Fig. 4F, Supplementary Table 4C).

### *USP15* Haploinsufficiency Is Associated With Reduced Progenitor TF Activity, While Complete Loss Shows Transcriptional Reprogramming

**Fig. 5:**
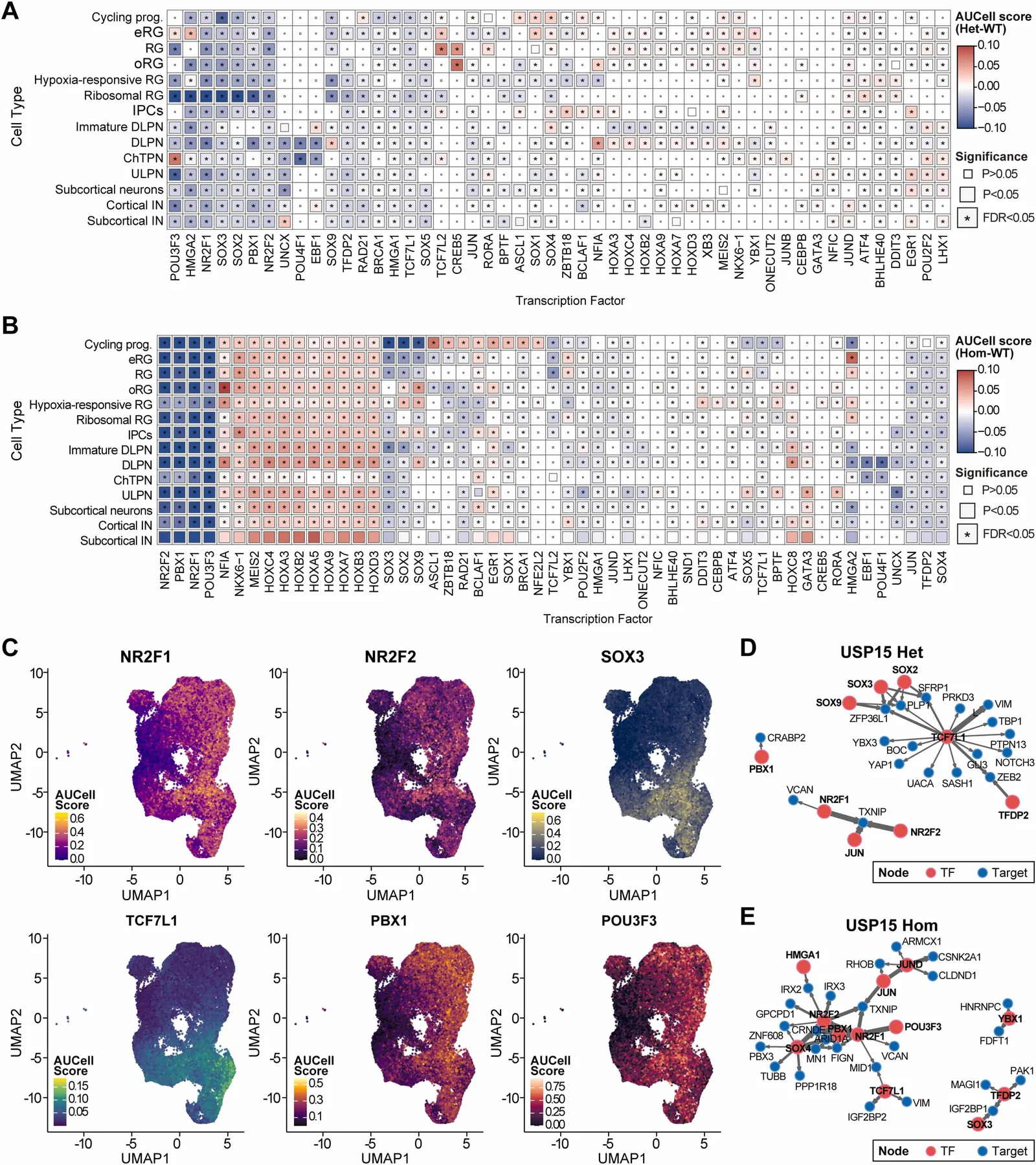
*Genotype-dependent dysregulation of transcription factor regulon activity in USP15 mutant organoids*. **(A-B)** Heatmap illustrating transcription factor regulon activity (median AUCell score) differences between Het and WT **(A)** and Hom and WT **(B)** across cell types. Red and blue indicate increased or decreased activity in the indicated mutant genotype relative to WT. Asterisks (*) denote statistical significance (FDR < 0.05, Wilcoxon rank-sum test). **(C)** UMAP visualization of AUCell scores for *NR2F1*, *NR2F2*, *SOX3*, *TCF7L1*, *PBX1*, and *POU3F3* regulons. Color intensity reflects regulon activity at the single-cell level. **(D-E)** Regulatory network illustrating relationships between TFs and their target genes based on inferred TF-target interactions and overlap with downregulated DEGs. TF-target associations were visualized when consistently observed in at least 5 cell types for USP15 Het and in at least 7 cell types for USP15 Hom. Edge thickness indicates the number of cell types in which the overlap was detected.

To delineate genotype-dependent effects of *USP15* loss, we profiled AUCell-based TF regulon activity across cell types (Fig. 5A, B). Het showed a widespread attenuation of TF activity relative to WT, most prominently in early radial glia and other progenitors (Fig. 5A, Supplementary Table 5A). Key cortical-progenitor regulators, including *POU3F3* (*BRN1*), *HMGA2*, *SOX2*, *PBX1*, *NR2F1*, *NR2F2*, and *SOX3*, were significantly reduced (FDR <2.60×10^−23^), while their spatial expression domains remained largely preserved on UMAP (*NR2F1*, *NR2F2*, and *SOX3*) (Fig. 5C). This suggests that *USP15* haploinsufficiency is associated with weaker activity states without relocating cell-type territories, together with reduced programs for stemness and early neurogenesis. Regulon-DEG overlaps were consistent with this pattern. This highlighted a recurrent *TCF7L1*-*V*IM  axis (11 cell types, progenitor-enriched) and a progenitor-restricted *ZFP36L1* module linked to SOX2, SOX3, and *TCF7L1* (7 cell types) (Fig. 5D). Given that *TCF7L1* is a WNT-linked regulator implicated in balancing progenitor proliferation and differentiation and that *ZFP36L1* controls mRNA-decay programs that influence fate timing, these overlaps are consistent with shifts in progenitor control of balance and timing. Additional overlaps connected *TXNIP* with *NR2F1*, *NR2F2*, and *JUN* (11 cell types), suggesting changes in stress- and redox-associated regulation. We further compared TF gene expression with regulon activity for SOX2, NR2F1, and NR2F2 and examined their associations with stress module scores. SOX2 regulon activity was reduced in Het despite unchanged SOX2 expression (median ΔmRNA = 0.000; median ΔAUCell = −0.020), whereas NR2F1 and NR2F2 showed reductions in both gene expression and regulon activity (Supplementary Table 5B). After correcting for cell-cycle state and detection rate, inverse associations between these TF regulons and stress modules were observed only in selected cell-type-stress module combinations, without a consistent stress-associated pattern (Supplementary Fig. 7, Supplementary Table 5C). Overall, Het exhibited widespread attenuation of progenitor TF regulon activity without global relocation of cell-type identities, echoing features reported in ASD-linked networks.

By contrast, in Hom, TF regulon activity was not simply reduced but reconfigured (Fig. 5B, Supplementary Table 5D). *HOX*-family regulons and stress-responsive regulators (*ATF4*, *DDIT3*, and *CEBPB*) showed increased regulon activity, whereas cortical-identity factors (*NR2F1/2, POU3F3*) and *PBX1* showed decreased activity across multiple cell types. This reprogramming was also reflected in regulon-DEG overlap analysis, which revealed genotype-dependent shifts in regulatory associations (Fig. 5D, E). *TCF7L1*-linked progenitor-maintenance overlaps that were prominent in Het were reduced in Hom. Progenitor-maintenance overlaps involving *ZFP36L1* were lost, and *TCF7L1*-linked overlaps were weakened in Hom. In Hom, overlaps between cortical-identity regulons (*POU3F3, PBX1*) and downregulated DEGs were more frequent, and overlaps involving *TXNIP* with the *NR2F1*, *NR2F2*, and *JUN* regulons were detected in both genotypes but occurred more often in Hom. Thus, Hom showed a distinct transcriptional program characterized by *HOX*-family activation and stress-response activity, rather than broad attenuation.

Given the increased *HOX*-family regulon activity in Hom, we next examined whether this signal corresponded to coordinated posteriorization. Regional identity analysis showed no consistent posterior shift across Hom progenitor subtypes. In *HOX*-high versus *HOX*-low DE analysis, canonical forebrain TFs, including *FOXG1, OTX2, SIX3, EMX1/2, LHX2*, and *FEZF2*, were not significantly reduced, whereas *PAX6* was significantly increased in *HOX*-high eRG (log2FC = +0.663, FDR = 2x10^−24^). HOX-positive Hom cells showed non-collinear co-expression of HOX paralogs (Spearman *r* = −0.249, *P* = .028). *HOX*-stress coupling analysis showed negative or nonsignificant associations between *HOX* activity and ER stress, oxidative stress, heat shock, and DNA damage programs after cell-cycle and detection-rate correction. These results indicate broad aberrant *HOX*-family activation rather than coordinated posteriorization or stress-associated regional identity disruption (Supplementary Fig. 8, Supplementary Table 5E-I).

### Convergence With ASD and NDD Genetics and ASD Mouse Signatures

**Fig. 6:**
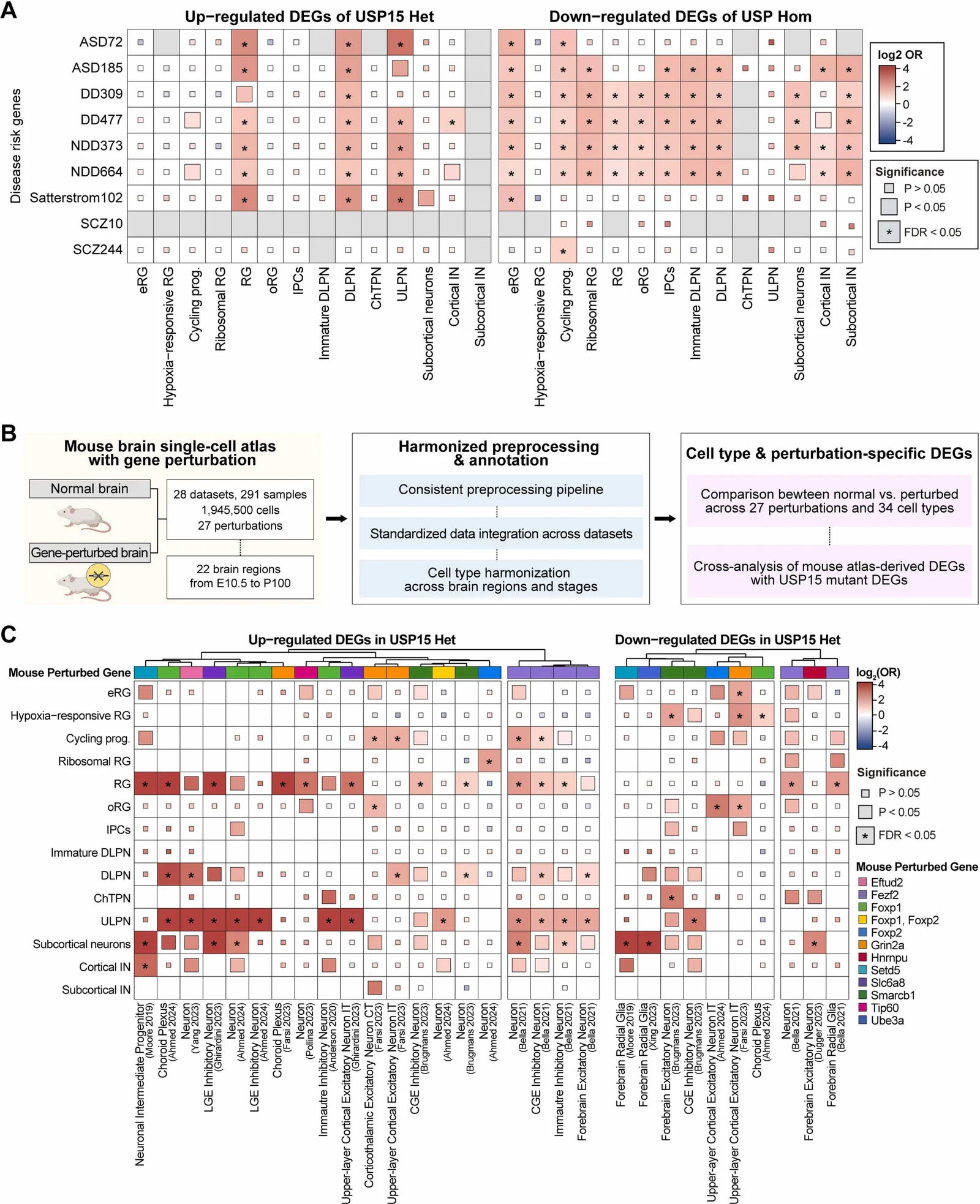
*Enrichment of DEGs in USP15 Het and Hom compared with known NDD risk genes and ASD mouse model DEGs.***(A)** Enrichment heatmap showing the overlap of cell-type DEGs from *USP15* Het upregulated DEGs (left) and Hom downregulated DEGs (right) with previously reported ASD-/NDD-associated disease risk gene sets. Gray boxes indicate comparisons with no overlapping genes. **(B)** Schematic workflow of comparison analysis using the mouse brain single-cell atlas with gene perturbations. **(C)** Hierarchical clustering heatmap comparing DEGs of *USP15* Het profiles across organoid cell types with established ASD mouse atlas datasets.

To assess the disease relevance of *USP15*-dependent programs, we compared cell-type-specific DEGs from *USP15* mutant organoids with curated ASD and NDD gene sets (Fig. 6A, Supplementary Table 6A-D). In deep-layer projection neurons from Het, upregulated DEGs were enriched for the ASD risk gene set (ASD72; FDR = 2.1x10^−2^), including synaptic and channel components (*GRIN2B*, *NRXN1*, *KCNQ3*, and *KCNMA1*) as well as transcriptional regulators (*MEIS2*) and neurodevelopmental signaling factors (*IRF2BPL*). A broader and more significant enrichment was observed for the established NDD gene set (FDR = 2.3x10^−3^), including TFs (*NFIX*, *NFIA*, *NFIB*, *TCF4*, *MYT1L*, and *BCL11B*), receptors (*GRIA2* and *GABBR2*), and developmentally relevant effectors (*NTRK2*, *SMARCA2*, *PRR12*, *DOT1L*, *PIK3R1*, *PRKCE*, and *CAMK4*) (Supplementary Table 6A, B). In deep-layer neurons of Hom, downregulated DEGs were likewise enriched for the established NDD gene set (NDD664; FDR = 5.4x10^−5^), including TFs (*PBX1*, *PBX3*, *ARID1A*, *RUNX1T1*, *KLF7*, and *SKI*), cytoskeletal and migration factors (*DPYSL2*), synaptic proteins (*SNAP25*), and signaling kinases (*CSNK1E*, *CSNK2A1*, *PAK1*, *ABL1*, and *CBL*) (Supplementary Table 6C, D). Notably, the overlap between upregulated genes in Het and downregulated genes in Hom was minimal, with only *BCL11B in common*, supporting genotype-specific, transcriptionally divergent responses during corticofugal neuron development.

We next examined whether progenitor compartments show similar convergence. In Hom cycling progenitors, upregulated DEGs were strongly enriched for the established NDD genes (FDR = 1.4x10^−6^), encompassing *NFIB*, *NFIA*, *EBF3*, *ELAVL3*, *KIF1A*, *SYT1*, *BCL11B*, *NOTCH1*, and *FOXP2*, consistent with broad disruption of signaling, transcriptional control, and neurogenic progression. Cross-compartment comparisons showed few overlaps between Hom cycling-progenitor upregulated sets and deep-layer neuron-downregulated sets (only *ELAVL4* and *BCL11A*). Still, substantial sharing with downregulated DEGs in cortical interneurons (*CSNK2A1*, *DPYSL2*, *PBX1*, *ARID1A*, *CSNK1E*, *WDR48*, *CELF2*, and *CTNNA2*) indicates that *USP15* insufficiency engages common developmental axes across distinct neuronal lineages while producing directionally different outcomes by cell type and genotype.

To place *USP15* within established ASD-related transcriptional programs, we compared DEG profiles between WT and *USP15* Het organoids with gene expression changes reported in the mouse single-cell atlas with ASD-associated gene perturbations (Fig. 6B). We found that *USP15* heterozygote DEGs showed notable overlap with those identified in *Fezf2*- and *Foxp1* mutant projection neurons for the ASD-relevant mouse models (Fig. 6C, Supplementary Table 6E, F). These 2 canonical ASD models are involved in corticofugal identity and the development of projection neurons (Chen et al., 2008, Srinivasan et al., 2012, Tantirigama et al., 2016, Usui et al., 2017). These overlaps suggest that the *USP15* mutation is associated with transcriptional programs also altered in ASD-related models, particularly those related to synaptic and projection neuron development.

## DISCUSSION

Ubiquitin genes play essential roles in early brain development, and disruptions in components of the ubiquitin-proteasome system affect the maintenance of neural progenitors, neuronal differentiation, and synaptic development. Genetic mutations in ubiquitin genes have long been reported in individuals with ASD (Amer-Sarsour et al., 2021, An et al., 2014, Cheon et al., 2019, Jolly and Kumar, 2022, Kasherman et al., 2020), including genes encoding E3 ligases (*UBE3A* (Khatri and Man, 2019, Xu et al., 2018, Roy et al., 2023), *HUWE1* (Cerminara et al., 2021; Moortgat et al., 2018; Nava et al., 2012)), deubiquitinating enzymes (*USP7* (Fountain et al., 2019, Hao et al., 2015, Kon et al., 2011), *USP9X* (Johnson et al., 2020; Reijnders et al., 2016), and proteasome subunits (*PSMD12* (Khalil et al., 2018; Küry et al., 2017)). Multiple deubiquitinating enzymes, including *USP7*, *USP9X*, *USP11*, and *OTUD7A*, have been identified with mutations in NDDs featuring autism-related phenotypes (Bridges et al., 2017, Chiang et al., 2021, Fountain et al., 2019, Kon et al., 2011, Premarathne et al., 2017, Stegeman et al., 2013, Uddin et al., 2018, Unda et al., 2023, Yin et al., 2018). Among these, our study provides a human organoid-based characterization of neurodevelopmental phenotypes associated with reduced USP15 dosage. We examined a heterozygous USP15 start-codon-disrupting variant (c.1A>T; p.Met1Leu) identified in an individual with ASD and characterized cellular and transcriptional differences in isogenic brain organoids carrying the variant. These findings provide a human organoid framework for characterizing neurodevelopmental alterations associated with reduced USP15 dosage in an ASD-relevant context.

*USP15* heterozygous organoids showed differences in progenitor-state maintenance and neuronal maturation-associated phenotypes. SOX2-positive progenitor fractions were comparable among WT, Het, and Hom organoids at day 60 but were reduced in Het organoids at days 80 and 100, consistent with reduced maintenance or representation of SOX2-positive progenitors at later time points. Het organoids also showed reduced deep-layer neuron markers and altered synaptic phenotypes at day 100. Thus, the Het phenotype involved both reduced progenitor-state maintenance and altered projection neuron-associated features. Reduced SOX2, NR2F1, and NR2F2 regulon activities in Het were not accompanied by a consistent stress-associated inverse pattern, and SOX2 regulon attenuation occurred despite unchanged SOX2 expression. These findings are more consistent with attenuation of progenitor regulatory programs over a generalized stress-response explanation. Genes altered in Het organoids significantly overlapped with established ASD risk genes (*TCF4*, *MYT1L*, *BCL11B*, *GRIN2B*, and *NRXN1*), placing the observed cellular and transcriptional features within an ASD-relevant neurodevelopmental context.

Comparison with ASD-associated mouse perturbation models identified prominent overlap between the USP15 Het transcriptional profile and signatures associated with Fezf2 and Foxp1 perturbation, placing the USP15 Het phenotype in the context of cortical projection neuron development. *FEZF2* is a key determinant of corticofugal and subcerebral projection neuron identity within the *BCL11B-/CTIP2*-associated deep-layer program (Chen et al., 2008, Srinivasan et al., 2012), consistent with the Fezf2-USP15 Het overlap in glutamatergic synaptic maturation and membrane-excitability genes. FOXP1 has broader roles in cortical neurogenesis, neuronal differentiation, migration, and projection neuron development (Li et al., 2015, Park et al., 2023, Pearson et al., 2020), suggesting that the Foxp1-USP15 overlap reflects broader projection neuron developmental programs. These overlaps are consistent with previous studies implicating progenitor and deep-layer projection neuron programs in ASD-related neurodevelopment (Willsey et al., 2013).

In Het deep-layer projection neurons, increased synaptic transcriptional programs occurred together with reduced PSD95 puncta density and SYN1-PSD95 colocalization, showing a mismatch between synaptic gene activation and puncta-level synaptic organization. This discordance was not explained by reduced DLG4/PSD95 or SYN1 transcript levels. In addition, bidirectional presynaptic SynGO enrichment, with upregulation of selected active-zone and vesicle-release-associated genes but downregulation of presynaptic ribosomal and local translation-related genes, suggests incomplete coordination of synaptic maturation programs. The current data do not, however, distinguish whether this mismatch reflects differences in protein abundance, trafficking, assembly, or other aspects of synaptic organization.

While both heterozygous and homozygous mutants exhibited reduced deep-layer neuron markers and altered synaptic puncta organization, homozygous mutants displayed distinct molecular changes compared with heterozygous mutants. At the transcriptional level, the 2 mutant states partially converged on USP15-sensitive programs, but the homozygous condition also showed additional complete-loss responses rather than a simple amplification of the heterozygous phenotype. Complete loss of *USP15* was associated with mitotic suppression, stress-response activation, and aberrant HOX-family activation, rather than a simple extension of the heterozygous phenotype.

The mitotic phenotype may help reconcile the apparent discrepancy between enlarged Hom organoids and reduced mitotic programs. Hom organoids showed an early increase in Ki67-positive progenitors at day 30, followed by later G1 enrichment, reduced mitotic activity, and persistence of viable but poorly proliferative progenitor populations without increased apoptotic cell loss. This sequence may contribute to the larger overall size of Hom organoids despite later suppression of active mitotic programs. In addition, Hom progenitors showed elevated cellular stress and senescence-associated programs, together with reduced SASP scores, supporting a viable but low-proliferative progenitor state rather than apoptotic cell loss. Finally, additional regional identity analyses supported a conservative interpretation that aberrant HOX-family gene expression in Hom progenitors does not represent coordinated posteriorization and is not simply explained by stress-high progenitor states. Instead, broad HOX-family activation and stress-associated programs appear to be separable transcriptional features associated with complete USP15 loss. Together, these findings suggest that USP15 dosage is associated with coordinated differences in progenitor cell-cycle states, neuronal maturation-associated programs, and synaptic organization.

Several methodological considerations should be noted. The scRNA-seq dataset comprised one pooled sample per genotype generated from 6 organoids within a single differentiation batch. This pooling strategy provided broad cell-type coverage and cell-state resolution, while organoid-to-organoid and batch-to-batch variability remains to be examined in independently differentiated samples. Such studies will further assess the reproducibility of the genotype-associated transcriptional patterns identified here. Synaptic puncta ROIs were selected according to predefined radial-position and image-quality criteria, and identical automated analysis parameters were applied across genotypes. Although ROI selection was not formally blinded to genotype, these standardized sampling and analysis procedures were used to minimize variability across conditions.

Our findings show that brain organoids carrying the USP15 variant exhibit genotype-associated molecular and cellular phenotypes relevant to early neurodevelopment. This study provides a human neural model for investigating how reduced USP15 dosage may influence developmental programs implicated in ASD. The transcriptomic and imaging findings also revealed complex relationships between gene expression and cellular phenotypes, including increased synaptic transcriptional programs alongside reduced puncta-level synaptic organization. Because USP15 is a deubiquitinating enzyme, future biochemical substrate assays, post-translational modification proteomics, protein localization analyses, and targeted perturbation or rescue experiments will be important for defining the mechanisms linking reduced USP15 dosage to the observed progenitor, neuronal, and HOX/stress-associated phenotypes.

## Author Contributions

T.-H.P., I.G.K., and S.Y.S. contributed equally to this work. J.K., J.-Y.A., and J.Y.L. are co-corresponding authors. Conceptualization, T.-H.P., I.G.K., S.Y.S., J.E.K., J.H.H., G.B., H.J.Y., J.K., J.-Y.A., and J.Y.L.; Methodology, T.-H.P., I.G.K., S.Y.S., J.E.K., J.K., J.-Y.A., and J.Y.L.; Software, I.G.K., H.P., and J.-Y.A.; Validation, T.-H.P., I.G.K., S.Y.S., H.P., J.E.K., S.L., Y.J.L., H.K., and J.-Y.A.; Formal analysis, T.-H.P., I.G.K., S.Y.S., H.P., J.E.K., S.L., and J.-Y.A.; Investigation, T.-H.P., I.G.K., S.Y.S., H.P., J.E.K., S.L., Y.J.L., H.K., J.H.H., G.B., H.J.Y., and J.-Y.A.; Resources, J.H.H., G.B., H.J.Y., J.K., J.-Y.A., and J.Y.L.; Data curation, T.-H.P., I.G.K., S.Y.S., and J.-Y.A.; Writing - original draft, T.-H.P., I.G.K., S.Y.S., J.K., J.-Y.A., and J.Y.L.; Writing - review and editing, T.-H.P., I.G.K., S.Y.S., H.P., J.E.K., S.L., Y.J.L., H.K., J.H.H., G.B., H.J.Y., J.K., J.-Y.A., and J.Y.L.; Visualization, T.-H.P., I.G.K., S.Y.S., H.P., J.E.K., S.L., and J.-Y.A.; Supervision, J.K., J.-Y.A., and J.Y.L.; Project administration, J.K., J.-Y.A., and J.Y.L.; Funding acquisition, H.J.Y., J.K., J.-Y.A., and J.Y.L. All authors approved the final paper.

## Declaration of Competing Interests

The authors declare that they have no known competing financial interests or personal relationships that could have appeared to influence the work reported in this paper.

## Footnotes

Appendix A Supplementary data associated with this article can be found in the online version at doi:10.1016/j.mocell.2026.100387.
