# Supplementary material for: USP15 mutation associated with autism spectrum disorder alters progenitor fate and neuronal maturation in human brain organoids
Source: Mol Cells. 2026 Jul 29;49(9):100387. doi: 10.1016/j.mocell.2026.100387 (PMC13499480; doi:10.1016/j.mocell.2026.100387)
Supplement: Supplementary file 1 [file mmc1.docx]

**Supplementary Information**

**Supplementary Figures**

**Supplementary Figure 1.** Gene expression patterns of USP genes across cell types in the developing human brain atlas

**Supplementary Figure 2.** Confirmation of USP15 Met1Leu mutagenesis

**Supplementary Figure 3.** Sanger sequencing analyses of off-targets

**Supplementary Figure 4.** UMAP visualization of a single-cell RNA-seq dataset of USP15 organoids and cell type annotation predicted by the developing human brain

**Supplementary Figure 5.** Cell-cycle distribution, apoptosis, and stress-associated transcriptional programs in USP15 mutant progenitors

**Supplementary Figure 6.** Lineage inference and genotype-wise trajectory comparison in USP15 mutant organoids

**Supplementary Figure 7.** Association between progenitor SOX2, NR2F1, and NR2F2 TF regulon activity and cellular stress programs

**Supplementary Figure 8.** Regional identity and stress-coupling analyses of HOX-family activation in USP15 Hom progenitors.

**Supplementary Figures**

**Supplementary Figure 1.** Gene expression patterns of USP genes and USP15 gene expression across cell types in the developing human brain atlas

(A) Heatmap of ubiquitination and USP gene expression (log2-normalized) across cell types of the developing human brain cell atlas from Kim et al. (B) USP15 gene expression across cell types and developmental trajectory in the developing human brain.

**Supplementary Figure 2.** Confirmation of USP15 Met1Leu mutagenesis

(A) Sanger sequencing confirms the A->T transversion (arrow) in heterozygote and homozygote clones leading to Met1Leu mutation. Note the presence of both A and T peaks in heterozygote. (B) Immunoblotting showing reduction and ablation of USP15 in heterozygote and homozygote respectively. α-tubulin is used as a loading control. (C) RT-qPCR assay result showing relative mRNA levels of USP15 in WT, Het, and Hom hiPSC lines (n=3). Data are presented as relative fold change. Error bars represent the range calculated by 2^−ΔΔCt±SEM^. *p < 0.05; **p < 0.01; ***p < 0.001(One-way ANOVA with Holm-Sidak’s post hoc test)

**Supplementary Figure 3.** Sanger sequencing analyses of off-targets.

Six genomic loci containing three mismatches were PCR-amplified, and sequences were analyzed by Sanger sequencing. Mismatch nucleotides are shown in lower-case letters. The chromosome and candidate sequence locations are specified. Oligonucleotide primers used for PCR amplification are presented. Analyses were performed for both homozygous and heterozygous clones.

**Supplementary Figure 4. UMAP visualization of a single-cell RNA-seq dataset of USP15 organoids and cell type annotation predicted by the developing human brain**

(A) UMAP of unintegrated data colored by sample identity (left), percentage of mitochondrial gene expression per cell (middle), and percentage of ribosomal gene expression per cell (right). (B) UMAP of the integrated dataset, colored by predicted cell types, using the developing human brain from Bhaduri et al. (C) Bar plots showing the proportion of predicted cell types across samples. (D) Comparison of predicted cell types and final annotated cell types

**Supplementary Figure 5. Cell-cycle distribution, apoptosis, and stress-associated transcriptional programs in USP15 mutant progenitors**

(A) Violin plots showing S-phase and G2/M module scores across progenitor subtypes in WT, USP15 Het, and USP15 Hom organoids. Stacked bar plots show the proportion of cells assigned to G1, S, or G2/M phase within each progenitor subtype. Statistical significance was determined by two-sided Wilcoxon rank-sum tests with Benjamini-Hochberg correction. NS, not significant; *FDR < 0.05; **FDR < 0.01; ***FDR < 0.001; ****FDR < 0.0001. (B) Quantification of cleaved caspase-3-positive cells among DAPI-positive cells in WT, USP15 Het, and USP15 Hom organoids at days 30 and 100, normalized to WT mean at each time point. Organoids were analyzed from 3 independent differentiation batches at day 30 (WT n = 12, Het n = 14, Hom n = 13) and 2 independent differentiation batches at day 100 (WT n = 8, Het n = 9, Hom n = 9). Each dot represents one organoid. Data are presented as mean ± SD. Statistical significance was assessed by one-way ANOVA. (C) Heatmaps showing differences in module scores for apoptosis-, senescence-, senescence-associated secretory phenotype (SASP-), and stress-associated gene programs in USP15 Het and USP15 Hom relative to WT. Scores are shown for pooled progenitors and individual progenitor subtypes. Statistical significance was determined by two-sided Mann-Whitney U tests with Benjamini-Hochberg correction. ER, endoplasmic reticulum.

**Supplementary Figure 6. Lineage inference and genotype-wise trajectory comparison in USP15 mutant organoids**

(A) Differentiation trajectory diagram inferred from lineage analysis (B) UMAP of pseudotime progression for each of the five lineages identified in (A). For each panel, cells belonging to the highlighted lineage are colored by pseudotime. (C) UMAP of the imbalance score of each cell across all inferred lineages. (D) UMAP of the overall lineage structures (minimum spanning tree connecting clusters) overlaid on all cells, colored by sample.

**Supplementary Figure 7. Association between progenitor TF regulon activity and cellular stress programs.**

Bubble plots showing partial Spearman correlations between SOX2, NR2F1, and NR2F2 regulon activity and stress-associated module scores across cell types in WT, USP15 Het, and USP15 Hom organoids. Asterisks denote FDR significance levels after Benjamini-Hochberg correction. *FDR < 0.05; **FDR < 0.01; ***FDR < 0.001.

**Supplementary Figure 8. Regional identity and stress-coupling analyses of HOX-family activation in USP15 Hom progenitors**

(A) Spearman correlation between HOX regulon activity and anterior-posterior (AP) score across cell types in USP15 Hom organoids. Positive correlations indicate maintenance of anterior identity, whereas negative correlations indicate a posterior-shift tendency. (B) Difference in AP score between HOX-high and HOX-low cells in USP15 Hom organoids. Values to the left indicate a shift toward a non-forebrain-like state, whereas values to the right indicate a more forebrain-like state. (C) Spearman correlation between HOX regulon activity and anterior identity score across progenitor and neuronal cell types in USP15 Hom organoids. Positive correlations indicate maintenance of anterior identity, whereas negative correlations indicate reduced anterior identity. (D) Pairwise Jaccard similarity of HOX-family gene detection among HOX-positive cells in WT and USP15 Hom organoids. HOX genes are ordered by paralog group (PG) to assess whether HOX co-expression follows a collinear axial pattern. (E) Correlation between HOX regulon activity and stress-associated gene program scores in USP15 Hom organoids. Statistical significance was determined using Spearman correlation or group comparison tests with the Benjamini-Hochberg correction. *FDR < 0.05.
